# Biomimetic Lipopolysaccharide‐Free Bacterial Outer Membrane‐Functionalized Nanoparticles for Brain‐Targeted Drug Delivery

**DOI:** 10.1002/advs.202105854

**Published:** 2022-03-31

**Authors:** Haiyan Chen, Mengyuan Zhou, Yuteng Zeng, Tongtong Miao, Haoyuan Luo, Yang Tong, Mei Zhao, Rui Mu, Jiang Gu, Shudi Yang, Liang Han

**Affiliations:** ^1^ Jiangsu Key Laboratory of Neuropsychiatric Diseases Research, College of Pharmaceutical Sciences Soochow University Suzhou Jiangsu 215123 P. R. China; ^2^ National Engineering Research Centre of Immunological Products, Department of Microbiology and Biochemical Pharmacy, College of Pharmacy Third Military Medical University Chongqing 400038 P. R. China; ^3^ Suzhou Polytechnic Institute of Agriculture Suzhou 215008 P. R. China

**Keywords:** bacterial outer membrane, biomimetic, blood–brain barrier, drug delivery, nanoparticles

## Abstract

The blood–brain barrier (BBB) severely blocks the intracranial accumulation of most systemic drugs. Inspired by the contribution of the bacterial outer membrane to *Escherichia coli* K1 (EC‐K1) binding to and invasion of BBB endothelial cells in bacterial meningitis, utilization of the BBB invasion ability of the EC‐K1 outer membrane for brain‐targeted drug delivery and construction of a biomimetic self‐assembled nanoparticle with a surface featuring a lipopolysaccharide‐free EC‐K1 outer membrane are proposed. BBB penetration of biomimetic nanoparticles is demonstrated to occur through the transcellular vesicle transport pathway, which is at least partially dependent on internalization, endosomal escape, and transcytosis mediated by the interactions between outer membrane protein A and gp96 on BBB endothelial cells. This biomimetic nanoengineering strategy endows the loaded drugs with prolonged circulation, intracranial interstitial distribution, and extremely high biocompatibility. Based on the critical roles of gp96 in cancer biology, this strategy reveals enormous potential for delivering therapeutics to treat gp96‐overexpressing intracranial malignancies.

## Graphical abstract

Biomimetic self‐assembled nanoparticles with a surface featuring a lipopolysaccharide‐free bacterial outer membrane are constructed to have blood–brain barrier crossing ability and for systemic drug delivery to the brain, based on the interactions between outer membrane protein A and gp96 on brain microvascular endothelial cells.

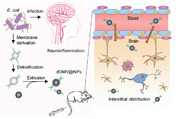

## 1 Introduction

The blood–brain barrier (BBB), a neurovascular system consisting of endothelial cells, pericytes, and astrocytes,^[^ ^1^ ^]^ strongly protects the brain by precisely regulating substance transport into the brain. For example, BBB‐specific tight junctions completely block paracellular diffusion through the paracellular space, while the low transcytosis rate in BBB endothelial cells severely limits transcellular vesicle transport.^[^ ^2^ ^]^ Only small hydrophobic molecules (molecular weight < ≈450) can use the transcellular diffusion pathway to penetrate the BBB. Other soluble nutrients (e.g., glucose, amino acids, and ferric ions in the form of holotransferrin) must be carried by specific transporters and receptors to enter the brain. Closure of the brain entry of most drugs directly leads to the failure of drug therapy for many brain diseases. It is urgent and vital to develop drug delivery systems that can efficiently transport drugs into the brain. Many efforts toward transporting drugs into the brain have been reported to date. One important kind of strategy is engineered nanoparticles (NPs) equipped with peptides capable of recognizing receptors (e.g., transferrin receptor and glucose transporter‐1) on BBB endothelial cells to initiate transcytosis or with functional molecules capable of opening tight junctions or inhibiting active efflux transport.^[^ ^3^ ^]^ However, after systemic administration, the accumulation rate of most engineered NPs in the brain does not exceed 1.0% dose per gram brain.^[^ ^4^ ^]^ Therefore, the NP‐based delivery strategy requires an innovative approach.

**Figure 1:**
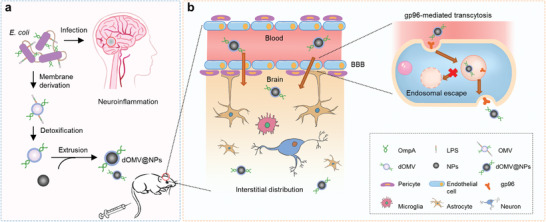
Design of EC‐K1 LPS‐free outer membrane‐coated dOMV@NPs for systemic drug delivery to the brain. a) Schematic representation of the brain entry of EC‐K1 to induce the occurrence of bacterial neuroinflammation and the use of a detoxified outer membrane from EC‐K1 to construct the brain‐targeted dOMV@NPs. b) In the blood vessels of the brain, dOMV@NPs are proposed to penetrate the BBB through OmpA‐gp96 interaction‐mediated transcytosis and are further distributed within the intracranial interstitial space.

Gram‐negative *Escherichia coli* K1 (EC‐K1) can cross the BBB and colonize the brain to induce bacterial meningitis inflammation (**Figure** **1**a). Outer membrane protein A (OmpA) is a 325 amino acid protein with 8 transmembrane domains and 4 extracellular loops and is an essential component of the bacterial outer membrane.^[^ ^5^ ^]^ gp96 (also known as GRP94) is an endoplasmic reticulum paralog of heat shock protein 90 that is not restricted to the endoplasmic reticulum and is also expressed at the surface of BBB endothelial cells.^[^ ^6^ ^]^ It has been extensively reported that surface‐exposed loops of OmpA interact with gp96 on BBB endothelial cells to initiate EC‐K1 binding to BBB endothelial cells and subsequent invasion.^[^ ^7^ ^]^ Moreover, the outer membrane proteins NlpI and IbeA promote EC‐K1 invasion of BBB endothelial cells.^[^ ^8^ ^]^ Therefore, the EC‐K1 outer membrane possesses the potential to mediate brain‐targeted drug delivery. Biomimetic drug delivery systems, e.g., cell membrane‐coated NPs, have attracted considerable attention owing to their ability to replicate highly complex but precise biological processes and have undergone rapid progression in terms of the state of the art.^[^ ^9^ ^]^ Nonreplicating secreted outer membrane vesicles (OMV) from gram‐negative bacteria have been successfully engineered as vaccines and tumor‐targeted drug delivery carriers.^[^ ^10^ ^]^ However, the use of the EC‐K1 outer membrane or OMV has not yet been reported for brain‐targeted drug delivery.

Inspired by the contribution of the bacterial outer membrane to EC‐K1 binding to and invasion of BBB endothelial cells and cell membrane coating nanotechnology (Figure 1a), we report a biomimetic strategy of utilizing the BBB invasion ability of the EC‐K1 outer membrane for brain‐targeted drug delivery. This strategy was implemented by biomimetic NPs, which were self‐assembled through the surface coating of parental NPs bearing terminal carboxyl groups with EC‐K1 lipopolysaccharide (LPS)‐free OMV (dOMV). The engineered dOMV@NPs were able to cross the BBB and enter the brain through the transcellular vesicle transport pathway, which was at least partially dependent on the interactions between OmpA and gp96 on BBB endothelial cells and OmpA‐mediated endosomal escape (Figure 1b). We further showed that biomimetic nanoengineering with an EC‐K1 LPS‐free outer membrane enabled long circulation and improved distribution in the intracranial interstitial space without concomitant toxic effects. In addition, owing to gp96 overexpression in brain metastatic cancer cells and its significant roles in the pathophysiological processes of brain metastases, this EC‐K1 LPS‐free outer membrane‐based biomimetic BBB‐penetrating strategy possesses enormous potential for delivering systemic therapy to treat both preclinical and clinical gp96‐overexpressing intracranial malignancies.

## 2 Results and Discussion

### 2.1 Active EC‐K1 was Observed in the Brains of Both Neonatal Rats and Adult Mice

EC‐K1 cells were tagged with green fluorescent protein (GFP) and bacterial luciferase (*luxCDABE*) using plasmid *pAKgfplux1* transfection through the electroporation technique.^[^ ^11^ ^]^ This plasmid tag was previously reported to be relatively stable in the absence of antibiotics and can be used for short‐term applications.^[^ ^11^ ^]^ Reporter expression is constitutive without the need for isopropyl *β*‐D‐thiogalactoside (IPTG) induction. The expressed GFP and bacterial luciferase do not require the addition of any exogenous substrates or cofactors to be functional but need active bacterial metabolism to provide the metabolic oxygen and energy requirements for *luxCDABE*. Therefore, tagging using the plasmid *pAKgfplux1* can be used for both in vitro and in vivo short‐term tracking of active bacteria. Under an IVIS imaging system, labeled bacteria were found to be able to emit apparent bioluminescence signals (Figure S1a, Supporting Information). GFP was also expressed and functional, as evidenced by flow cytometry and fluorescence microscopy (Figure S1b, Supporting Information). A linear relationship existed between the bioluminescence signal intensity and the number of bacteria (Figure S1c, Supporting Information).

Multiple administration routes, e.g., intracardiac injection, intravenous injection, intraperitoneal injection, and oral administration, have been proven capable of achieving a significant accumulation of EC‐K1 in the brain.^[^ ^12^ ^]^ Intracardiac injection and subcutaneous injection were shown to produce similar bacteremia levels (sufficient for brain entry of circulating EC‐K1) and meningitis rates.^[^ ^12d^ ^]^ For neonatal rats, intravenous injection is difficult because of the vagueness of the tail vein. The technique of intracardiac injection is mature, routine, and often used to establish animal models of brain metastases.^[^ ^13^ ^]^ We then investigated the brain entry of EC‐K1 after intracardiac injection. For neonatal rats, the signal intensity from transformed EC‐K1 in the brain was significantly higher than that in peripheral organs at 12 h after intracardiac injection of low‐dose bacteria (1 × 10^3^ colony forming units (CFU); Figure S1d, Supporting Information). For adult mice, a significant bacterial bioluminescence signal in the brain was shown only under conditions of both high‐dose bacterial injection (5 × 10^7^ CFU) and high bacterial signal intensity in blood and peripheral tissues. When the bacterial titer in blood and peripheral tissues was reduced by intravenously injecting the antibiotic gentamicin (15 mg kg^−1^), the bacterial bioluminescence signal in the brain was also significantly reduced. These data implied that EC‐K1 can cross both the immature BBB and adult BBB to enter the brain. However, high concentrations of bacteria in the blood may be needed to cross the adult BBB. These findings are consistent with the preferential occurrence of EC‐K1 meningitis in human neonates and the low incidence of EC‐K1 meningitis in human adults, which may be related to antibiotic use. Despite these findings, the nanoscale effect may endow the engineered dOMV@NPs with the ability to cross the BBB.

### 2.2 Design and Characterization of dOMV@NPs

**Figure 2:**
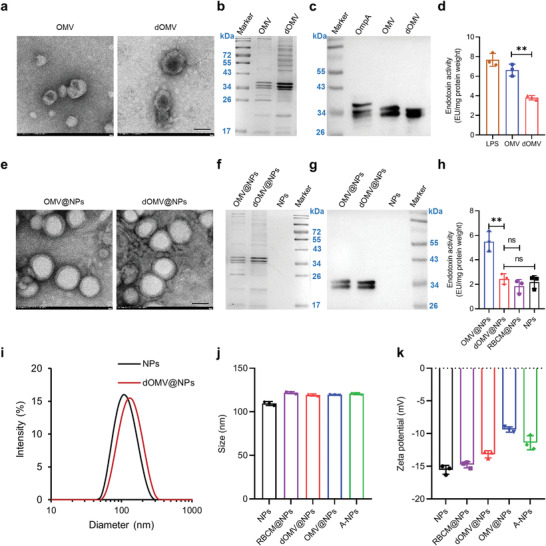
Characterization of dOMV and dOMV@NPs. a) Representative TEM images of OMV and dOMV. Scale bar, 50 nm. b,c) SDS–PAGE (b) and western blot (c) of OMV and dOMV. d) Endotoxin activity of OMV and dOMV was analyzed by LAL assay. e) Representative TEM images of OMV@NPs and dOMV@NPs. Scale bar, 50 nm. f,g) SDS–PAGE (f) and western blot (g) of OMV@NPs and dOMV@NPs. h) Endotoxin activity of OMV@NPs and dOMV@NPs was analyzed by LAL assay. i) Distribution of the hydrodynamic diameter of dOMV@NPs by dynamic light scattering. j) The mean hydrodynamic diameter of the indicated NPs. k) The zeta potential of the indicated NPs by dynamic light scattering. Error bars represent the SD (*n* = 3). ns (not significant), **p < 0.01, one‐tailed unpaired *t*‐test.

Both dOMV and OMV were extracted and purified from EC‐K1 overnight culture (OD_600_ ≈1.5) according to previously described methods.^[^ ^14^ ^]^ Compared with that for OMV, the extraction and purification processes for dOMV not only remove LPS but also increase vesicle release and are traditionally used to prepare vaccines with reduced toxicity.^[^ ^14a^ ^]^ The average yield of dOMV (2.0 mg per liter bacterial fluid) was 3.48 times that of OMV (0.58 mg per liter bacterial fluid, Figure S2, Supporting Information). Under transmission electron microscopy (TEM), both dOMV and OMV displayed irregular ellipsoidal structures with diameters of dozens of nanometers (**Figure** **2**a). As shown by sodium dodecyl sulfate polyacrylamide gel electrophoresis (SDS–PAGE, Figure 2b), there were some differences in protein components between dOMV and OMV, which is ascribed to their different origins (bacterial outer membrane and bacterial culture supernatant, respectively). However, OmpA was found in both dOMV and OMV (Figure 2c). Owing to the absence of LPS, dOMV were found to have reduced endotoxin activity compared with that of free LPS and OMV (Figure 2d). Because LPS induces hemolysis in human sepsis,^[^ ^15^ ^]^ dOMV also showed almost no hemolysis compared with LPS and OMV (Figure S3, Supporting Information).

Parental NPs with surface terminal carboxyl groups were prepared using poly(lactic‐co‐glycolic acid) (PLGA) as the starting material through the emulsion solvent evaporation method as we described previously.^[^ ^13^ ^]^ Both dOMV and OMV were transferred to the surface of parental NPs via the extrusion method (200 nm membrane) to fabricate biomimetic dOMV@NPs and OMV@NPs. Under TEM, compared with their original blank vesicles, dOMV@NPs and OMV@NPs displayed more regular spheres with noticeable core‐shell structures and diameters of approximately one hundred nanometers (Figure 2e). SDS–PAGE and western blot data indicated good retention of membrane proteins (especially OmpA) on biomimetic dOMV@NPs and OMV@NPs (Figure 2f,g). Similar to the absence of LPS in purified dOMV, dOMV@NPs exhibited much lower endotoxin activity than OMV@NPs (Figure 2h). Red blood cell membrane (RBCM)‐coated NPs (RBCM@NPs) and brain‐targeted angiopep‐2‐modified NPs (A‐NPs^[^ ^13a^ ^]^) were used as controls. The endotoxin activity of the dOMV@NPs was comparable to that of the parental NPs and RBCM@NPs. In addition, the hemolysis induced by dOMV@NPs was significantly lower than that induced by OMV@NPs and comparable to that of parental NPs, RBCM@NPs, and A‐NPs (Figure S4, Supporting Information), which was consistent with the previous results of OmpA‐modified magnetite nanobioconjugates.^[^ ^16^ ^]^ With a Gaussian distribution (Figure 2i), the median hydrodynamic diameter of dOMV@NPs (119.3 nm) was comparable to that of OMV@NPs (119.5 nm), RBCM@NPs (121.9 nm), and A‐NPs (120.8 nm) and slightly higher than that of parental NPs (109.5 nm, Figure 2j). Compared with that of parental NPs (−15.5 mV), the absolute value of the negative zeta potential of dOMV@NPs decreased (−13.2 mV) owing to the dOMV coating (Figure 2k). Notably, the zeta potential of dOMV@NPs was more negative than that of OMV@NPs (−9.4 mV), which may be due to the replacement of LPS with negatively charged deoxycholate in dOMV.^[^ ^14b^ ^]^ These results confirmed the successful membrane coating on the surface of the parental NPs.

### 2.3 Interactions Between OmpA and gp96 Endow dOMV@NPs with the Ability to Cross the BBB

**Figure 3:**
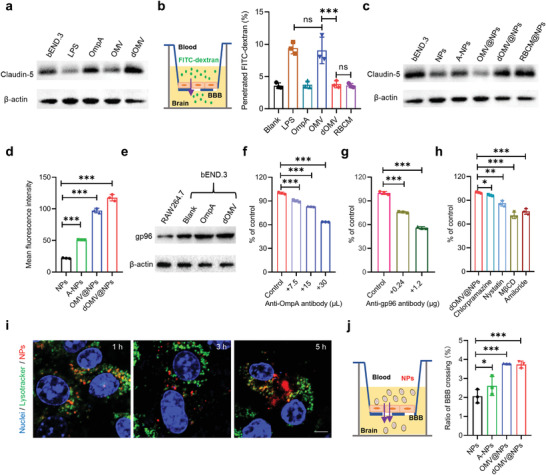
dOMV@NPs penetrate the BBB through the OmpA‐gp96 interaction‐mediated transcellular vesicle transport pathway. a) Levels of claudin‐5 on bEND.3 BBB endothelial cells after stimulation with the indicated bacterial components (20 µg mL^−1^ protein or 2.24 ng mL^−1^ LPS for 24 h) were analyzed by western blot. b) Paracellular penetration of 40 kDa FITC‐dextran (1 mg mL^−1^, 6 h incubation) across the in vitro BBB (which was prestimulated with the indicated bacterial components at 20 µg mL^−1^ protein or 2.24 ng mL^−1^ LPS for 12 h). c) Claudin‐5 expression on bEND.3 cells after stimulation with the indicated NPs (20 µg ml^−1^ protein for 24 h) was analyzed by western blot. d) Cellular uptake of the indicated DiR‐labeled NPs (1.5 µg DiR mL^−1^ for 6 h) by bEND.3 cells was measured by flow cytometry. e) gp96 on RAW264.7 macrophages and bEND.3 cells stimulated with either OmpA or dOMV was analyzed by western blot. f–h) Cellular uptake of doxorubicin‐labeled dOMV@NPs (5 µg doxorubicin mL^−1^ for 1.5 h) by bEND.3 cells in the presence of anti‐OmpA antibody (f), anti‐gp96 antibody (g), or the indicated endocytosis inhibitors (h) was measured by flow cytometry. i) Intracellular localization of dOMV@NPs (12 µg doxorubicin mL^−1^ for 1 h) after further incubation with fresh medium for different times, imaged using a confocal laser scanning microscope. Scale bar, 5 µm. j) Penetration of the indicated doxorubicin‐labeled NPs (5 µg doxorubicin mL^−1^ for 6 h) across the in vitro BBB. Error bars represent the SD (*n* = 3). ns (not significant), *p < 0.05, **p < 0.01, ***p < 0.001, one‐tailed unpaired *t*‐test.

We first studied whether dOMV could open tight junctions in an in vitro BBB model (**Figure** **3**a). Claudins are major components of tight junctions, while claudin‐5 dominates BBB tight junctions by limiting paracellular diffusion.^[^ ^17^ ^]^ Claudin‐5 levels in bEND.3 BBB endothelial cells were maintained after treatment with either recombinant OmpA or dOMV but were reduced after incubation with either LPS or OMV. In addition, similar data were obtained for occludin and zonula occludens 1 (ZO‐1), two other markers of tight junctions, where neither OmpA nor dOMV changed the expression levels of occludin and ZO‐1 (Figure S5, Supporting Information). Then, the penetration of BBB‐impermeable fluorescein isothiocyanate‐dextran (FITC‐dextran, 40 kDa) through the in vitro BBB model was measured to evaluate the effects of these bacterial components on BBB paracellular permeability (Figure 3b). Neither recombinant OmpA nor dOMV altered the permeation efficiency of FITC‐dextran through compact‐monolayered bEND.3 cells on the Transwell insert, suggesting that they failed to open tight junctions in the BBB to trigger BBB paracellular crossing of coadministered agents or themselves. However, both LPS and LPS‐containing OMV significantly enhanced the permeation efficiency of FITC‐dextran across the in vitro BBB, suggesting that they can open BBB tight junctions to promote BBB paracellular transport. For NPs, OMV@NPs reduced claudin‐5 expression in bEND.3 cells, but dOMV@NPs did not (Figure 3c), which was similar to the effects of OMV and dOMV (Figure 3a). These data revealed that LPS‐free dOMV could not modulate BBB tight junctions and subsequent BBB paracellular permeability and may be able to avoid the side effects (e.g., edema, neuroinflammation, and cognitive impairment) associated with tight junction opening‐based brain‐targeted drug delivery strategies.

Next, BBB transcellular transport was investigated by assessing BBB endothelial uptake, endosomal escape, and BBB transcellular permeation efficiency. First, dOMV@NPs were shown to be internalized into bEND.3 cells with an efficiency higher than that of parental NPs, A‐NPs, and RBCM@NPs (Figure 3d and Figure S6, Supporting Information). According to the role of OmpA in BBB endothelial binding and invasion of EC‐K1 and its existence on both dOMV@NPs and OMV@NPs, the uptake of dOMV@NPs and OMV@NPs in bEND.3 cells may be strongly associated with the interactions between OmpA and its receptor gp96. The gp96 level in bEND.3 cells was higher than that in RAW264.7 macrophages (Figure 3e). More importantly, both recombinant OmpA and dOMV significantly enhanced the expression of gp96, which was consistent with previous reports where OmpA^+^ *E. coli* (EC‐K1) upregulated the expression of gp96 in both brain endothelial cells (through the nitric oxide/cGMP signaling pathway) and leukocytes, while OmpA^−^ *E. coli* did not affect gp96 expression.^[^ ^7^, ^18^ ^]^ The upregulated gp96 expression by OmpA and dOMV may be able to autocatalytically promote the brain‐targeted efficiency of dOMV@NPs. Furthermore, gp96 was found to be localized both inside the cytoplasm (endoplasmic reticulum) and at the plasma membrane in bEND.3 BBB endothelial cells (Figure S7, Supporting Information), confirming that gp96 may be able to trigger receptor‐mediated endocytosis. Both the anti‐OmpA antibody and anti‐gp96 antibody inhibited the uptake of dOMV@NPs in bEND.3 cells by 36.5% and 44.1% (Figure 3f,g), respectively, confirming the close involvement of the interactions between OmpA and gp96 in BBB endothelial uptake of dOMV@NPs. Lipid rafts (29.0%, M*β*CD), macropinocytosis (24.1%, amiloride), and caveolae (13.7%, nystatin) were involved in the internalization of dOMV@NPs (Figure 3h), which was consistent with previous reports on EC‐K1 invasion of BBB endothelial cells via the interactions of OmpA with gp96 present in caveolae.^[^ ^19^ ^]^ The internalized dOMV@NPs were first found to be localized with endosomes, further proving the role of gp96‐mediated endocytosis in the uptake of dOMV@NPs (Figure 3i). Then, significant endosomal escape was found, which was ascribed to OmpA‐mediated endosomal escape.^[^ ^16^ ^]^ Finally, in vitro BBB crossing of labeled dOMV@NPs was evaluated (Figure 3j). Approximately 3.73% of dOMV@NPs and 3.78% of OMV@NPs crossed the in vitro BBB and reached the abluminal side, which was significantly higher than that of parental NPs and A‐NPs, indicating that dOMV@NPs with transcellular BBB crossing ability can be used to mediate effective brain‐targeted drug delivery.

### 2.4 Macrophage Uptake, Pharmacokinetics, and Efficient Brain Accumulation of dOMV@NPs

**Figure 4:**
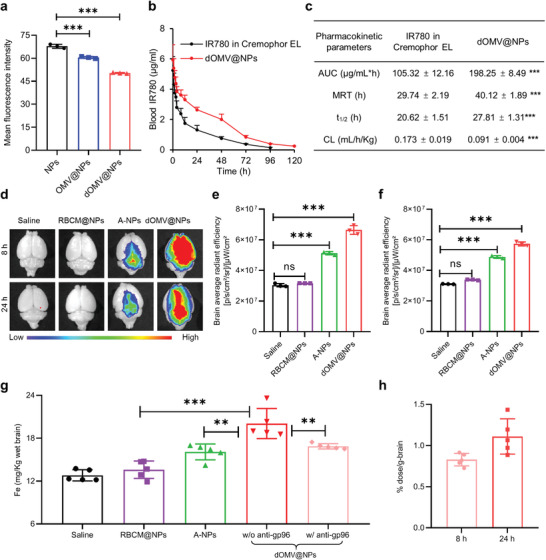
Pharmacokinetics and brain accumulation in normal mice. a) Cellular uptake of the indicated DiR‐labeled NPs (1.5 µg DiR mL^−1^ for 3 h) by RAW264.7 macrophages was measured by flow cytometry. b,c) Blood concentration‐time profiles (b) and pharmacokinetic parameters (c) in normal mice (*n* = 5). d–f) Brain accumulation of IR780 in normal mice at 8 and 24 h after injection of the indicated IR780‐labeled NPs was qualitatively imaged (d) and semiquantitatively analyzed (e,f) by measuring the fluorescence of IR780. g) Quantitative measurement of intracranial Fe concentration by ICP–MS at 8 h after intravenous injection of NPs loaded with SPIO (*n* = 5). h) The brains were further excised, and IR780 was extracted to quantitatively investigate the exact % ID/g brain (*n* = 5). Error bars represent the SD (*n* = 3, unless otherwise specified). ns (not significant), **p < 0.01, ***p < 0.001, one‐tailed unpaired *t*‐test.

Macrophage uptake was first investigated to predict in vivo blood behavior (**Figure** **4**a and Figure S8, Supporting Information). Both parental NPs and OMV@NPs were internalized by macrophages with high efficiency, which was ascribed to the high absolute value of the zeta potential of parental NPs^[^ ^20^ ^]^ and the pathogen‐associated molecular pattern on OMV@NPs (LPS), respectively. With a lower zeta potential and absence of LPS, dOMV@NPs showed less macrophage uptake than both OMV@NPs and RBCM@NPs (Figure 4a and Figure S8, Supporting Information), suggesting the stealth effect and possibly improved in vivo pharmacokinetics of dOMV@NPs. Pharmacokinetic studies were further performed to explore the blood circulation of dOMV@NPs (Figure 4b,c). Compared with that of IR780 in Cremophor EL and ethanol, the blood elimination of IR780‐labeled dOMV@NPs was delayed, with a significantly greater area under the curve (AUC_0→t_), longer mean residence time (MRT_0→t_), longer blood circulation half‐life (t_1/2_), and weaker clearance (CL). Notably, Cremophor EL has been shown to encapsulate drugs and delay clearance of loaded drugs (e.g., paclitaxel) by forming micelles, leading to plasma concentration profiles of loaded drugs even similar to those with albumin‐bound nanoparticles.^[^ ^21^ ^]^ The long blood circulation time of IR780 (t_1/2_ 20.62 h) may be due to its micellar encapsulation by Cremophor EL. Compared with that of IR780 in Cremophor EL, the longer blood circulation of dOMV@NPs (t_1/2_ 27.81 h) demonstrated the stealth effect of dOMV@NPs. In addition to the absence of LPS, the prolonged circulation of dOMV@NPs may be related to the escape from complement‐mediated bacterial killing. This escape was previously thought to be mediated by outer membrane protein NlpI‐facilitated interactions between OmpA and the complement regulator C4bp.^[^ ^22^ ^]^ These data suggested that dOMV@NPs could delay the elimination of the loaded drug from blood.

Encouraged by effective BBB penetration with an explicit mechanism and prolonged pharmacokinetics, we further evaluated whether dOMV@NPs could efficiently accumulate in the mouse brain (Figure 4d–h). Fluorescence images clearly revealed that the intracranial fluorescence signal intensity of dOMV@NPs was significantly higher than that of RBCM@NPs and A‐NPs in normal mice (Figure 4d and Figure S9, Supporting Information), indicating that dOMV@NPs were capable of efficiently penetrating through the intact BBB into the brain. The lack of accumulation of RBCM@NPs in the brain may be because RBCM could not significantly affect the BBB via just the phospholipid bilayer. Brain‐targeted A‐NPs also showed weak intracranial fluorescence signal intensity, which was ascribed to the low expression of the receptor LRP1 in BBB endothelial cells.^[^ ^23^ ^]^ Semiquantitative analysis showed that mice treated with RBCM@NPs showed very weak intracranial fluorescence intensity, which was comparable to that in the saline group (Figure 4e,f). At 8 h after NP injection, the increase in intracranial fluorescence intensity by dOMV@NPs and A‐NPs was 28.7‐fold and 16.6‐fold higher than that by RBCM@NPs (Figure 4e), respectively. At 24 h after NP injection, the difference in intracranial accumulation between the dOMV@NPs and RBCM@NPs became 9.5‐fold, while that between the A‐NPs and RBCM@NPs became 6.4‐fold (Figure 4f). The fluorescence intensity of dOMV@NPs in the brain was also significantly higher than that of A‐NPs, with differences of 1.73‐fold at 8 h and 1.48‐fold at 24 h. Another group of normal mice was intravenously injected with NPs loaded with superparamagnetic iron oxide (SPIO).^[^ ^24^ ^]^ At 8 h after injection, brains were excised, digested, and analyzed for Fe content by inductively coupled plasma‐mass spectrometry (ICP–MS) to quantify accumulated dOMV@NPs in the brain (Figure 4g). Compared to that in untreated mice, the intracranial Fe concentration in mice treated with RBCM@NPs and A‐NPs increased by 0.788 and 3.28 mg kg^−1^, respectively. The Fe concentration in the brain rose by 7.25 mg kg^−1^ in SPIO‐loaded dOMV@NP‐treated mice. The increased intracranial Fe concentration after treatment with dOMV@NPs and A‐NPs was 9.20‐fold and 4.16‐fold higher than that with RBCM@NPs. EC‐K1 invades BBB endothelial cells and enters the brain in a transcytosis manner.^[^ ^6^, ^25^ ^]^ The receptor gp96 has been extensively reported to be responsible for transcytosis of EC‐K1 across the BBB.^[^ ^7^, ^26^ ^]^ Pretreatment with the anti‐gp96 antibody significantly decreased the increase in intracranial Fe concentration induced by dOMV@NPs (Figure 4g), further demonstrating gp96‐mediated transcellular transport in the BBB crossing behavior of dOMV@NPs. The accumulation of dOMV@NPs in the brain was further accurately quantified and found to be 0.83% dose per gram brain at 8 h and 1.11% dose per gram brain at 24 h (Figure 4h). Most nanocarriers, even those engineered to enhance BBB penetration, have low brain accumulation rates (<1% dose per gram brain) after systemic administration.^[^ ^4^ ^]^ For example, the brain accumulation rate of a glucose‐modified nanocarrier was reported to be ≈0.3% dose per gram brain.^[^ ^3a^ ^]^ In another study, a biologically inspired apolipoprotein‐reconstituted high‐density lipoprotein nanostructure was reported to accumulate in the brain with an efficiency of ≈0.4% dose per gram brain.^[^ ^27^ ^]^ Therefore, the accumulation rate of 1.11% dose per gram brain is appealing because it is higher than those reported to date for many brain‐targeted drug delivery systems. Further optimization, e.g., size, surface potential, and functionalization, may be able to further improve the brain accumulation rate of dOMV@NPs. These observations demonstrated that the coating of dOMV was conducive to NPs passing through the intact BBB into the brain. Taken together, we believe that our comprehensive OMV‐mimetic strategy successfully conferred dOMV@NPs with superior BBB penetration and brain‐targeted ability.

### 2.5 Intracranial Microdistribution and Immunohistochemical Localization

**Figure 5:**
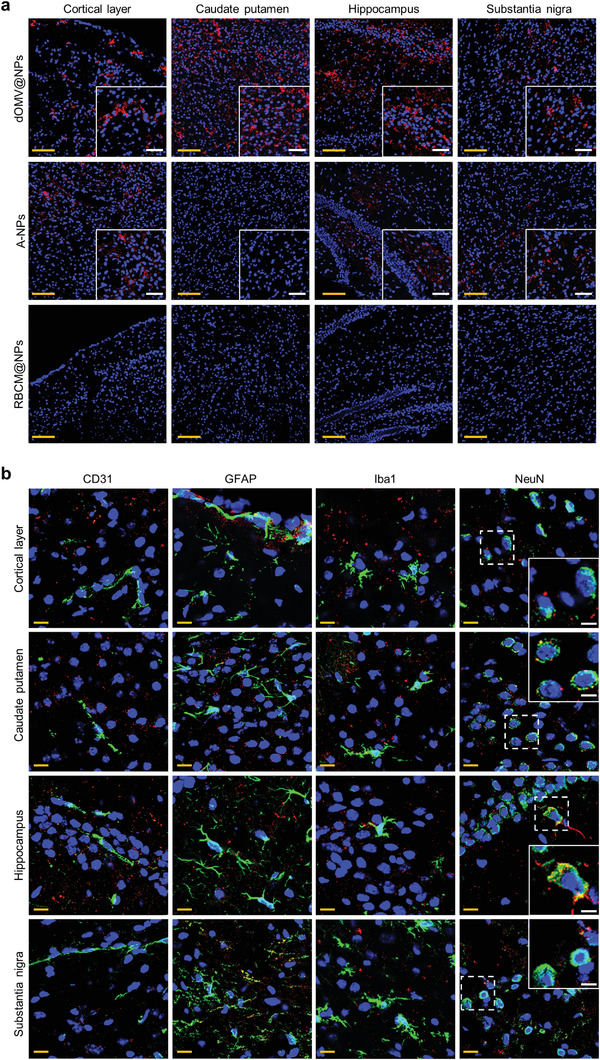
Intracranial microdistribution and immunohistochemical localization. a) Microdistribution of doxorubicin (red) in the indicated intracranial regions at 12 h after the second intravenous injection of the indicated doxorubicin‐labeled NPs into normal mice. Nuclei (blue) were stained with DAPI. Yellow scale bars and white scale bars (in insets) indicate 100 and 50 µm, respectively. b) Immunohistochemical analysis of cerebral sections for the indicated intracranial regions was performed to check the colocalization of dOMV@NPs with various types of intracranial cells. Cerebral sections obtained at 12 h after the second intravenous injection of doxorubicin‐labeled dOMV@NPs were stained with anti‐CD31, anti‐GFAP, anti‐Iba1, and anti‐NeuN antibodies (green) to label brain endothelial cells, astrocytes, microglia, and neurons, respectively. Nuclei (blue) were stained with DAPI. Yellow scale bars and white scale bars (in insets) indicate 20 and 10 µm, respectively.

Doxorubicin is often used as a model drug due to its broad anticancer activity and relatively stable fluorescence, which can be used for in vitro and in vivo tracking.^[^ ^28^ ^]^ In our previous work, doxorubicin was efficiently loaded by PLGA‐based NPs.^[^ ^13^ ^]^ After encapsulation by NPs, doxorubicin maintained a steady fluorescence intensity at room temperature for at least seven days (Figure S10, Supporting Information). Based on these advantages, doxorubicin was used to track the location of NPs in sliced brains. Under a confocal laser scanning microscope, while labeled RBCM@NPs were invisible in any brain region, labeled A‐NPs were observed in the prefrontal cortex, hippocampus, and substantia nigra but were almost nonexistent in the caudate‐putamen (**Figure** **5**a). However, labeled dOMV@NPs were observed in these four intracranial regions with an accumulation efficiency much higher than that of A‐NPs.

We further analyzed the immunohistochemical localization of dOMV@NPs in these four intracranial regions to determine the accurate intracranial destination of dOMV@NPs. RBCM@NPs were not recognized in the brain, and no colocalization with brain cells was found in any brain region according to immunohistochemical analysis (Figure S11, Supporting Information). Although A‐NPs were shown to be able to successfully enter the prefrontal cortex, hippocampus, and substantia nigra, no colocalization of A‐NPs with brain cells was found, except endothelial cells and microglia in the prefrontal cortex and astrocytes in either the hippocampus or substantia nigra (Figure S12, Supporting Information). Highly efficient intracranial accumulation of dOMV@NPs was further confirmed (Figure 5b). Endothelial entrapment is often shown in transferrin receptor‐mediated transportation owing to the low dissociation of receptor–ligand complexes.^[^ ^3^, ^29^ ^]^ The extremely low colocalization of dOMV@NPs with endothelial cells revealed that most dOMV@NPs had escaped endothelial entrapment and successfully crossed the BBB. EC‐K1 has been reported to invade hippocampal neurons.^[^ ^8b^ ^]^ In vitro immortalized HT22 mouse hippocampal neuronal cells were shown to express significant gp96 and to be able to internalize dOMV@NPs (Figure S13, Supporting Information). However, a previous report showed that less than 6% of primary rat hippocampal neurons were EC‐K1 positive after EC‐K1 infection.^[^ ^8a^ ^]^ In this study, most dOMV@NPs remained in the intracranial interstitial space and barely colocalized with brain cells, including neurons, in all brain regions. This finding was thus ascribed to the nonproliferative feature of intracranial neurons and implied that dOMV@NPs may be able to escape the side effects to normal brain cells when used to treat intracranial proliferative lesions.

dOMV@NPs may be used to treat malignant intracranial lesions. gp96 has been reported to play important roles in the physiological and pathological processes of brain metastases.^[^ ^30^ ^]^ Mouse grafts of breast cancer brain metastases derived from brain‐tropic 4T1‐BR5 murine mammary carcinoma cells and Her2‐overexpressing MDA‐MB‐231‐BR‐Her2 (231Br) human metastatic breast cancer cells have been well characterized to recapitulate the pathology of human breast cancer brain metastases.^[^ ^31^ ^]^ Based on these previous reports, we found high expression of gp96 in 4T1‐BR5 cells and 231Br cells (Figure S14, Supporting Information). Both cancer cell lines were shown to be able to significantly internalize dOMV@NPs (Figure S15, Supporting Information). With gp96 expression in cancer cells similar to or even higher than that in neurons, dOMV@NPs possess the potential to efficiently enter intracranial proliferative cancer cells rather than relatively quiescent neurons for specific cancer chemotherapy. Doxorubicin‐loaded dOMV@NPs were used to evaluate the in vivo targeting efficiency and therapeutic efficacy for breast cancer brain metastases in mice because of the broad anticancer activity and stable fluorescence of doxorubicin (Figure S10, Supporting Information). The expression of gp96 in BBB endothelial cells cultured in 231Br conditioned medium (brain metastatic tumor BBB) was comparable to that in normal BBB endothelial cells, leading to similar BBB penetration efficiency under both conditions (Figure S16a, Supporting Information). In the brain with GFP‐expressed 231Br metastatic breast cancer cells, doxorubicin‐loaded dOMV@NPs preferentially colocalized with tumor cells rather than normal brain cells (Figure S16b,c, Supporting Information), indicating the brain metastasis targeting efficiency of dOMV@NPs. The IC_50_ of doxorubicin‐loaded dOMV@NPs (0.27 µg mL^−1^) on 231Br cells after 24 h incubation was slightly higher than that of free doxorubicin (0.12 µg mL^−1^), which may be due to the release process of doxorubicin. Then, the in vivo therapeutic efficacy was evaluated by monitoring the survival of mice with breast cancer brain metastases over 30 days (Figure S16d, Supporting Information). Systemic treatment of doxorubicin‐loaded dOMV@NPs markedly lengthened the survival of mice bearing brain metastases. The median survival time of mice treated with saline and free doxorubicin was 24 and 28 days, respectively. The median survival time of mice treated with doxorubicin‐loaded dOMV@NPs was not yet detected, suggesting the significant therapeutic effect of doxorubicin‐loaded dOMV@NPs.

### 2.6 Negligible Effects on Physiological Function and the Immune System

**Figure 6:**
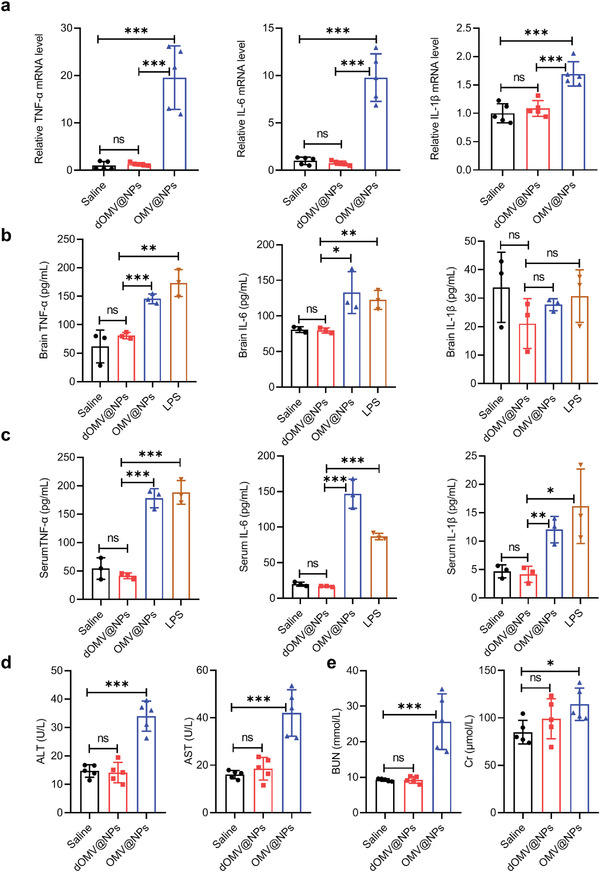
Biosafety assessment of dOMV@NPs. a) mRNA levels of TNF‐*α*, IL‐6, and IL‐1*β* in brains were quantitatively measured 24 h after intravenous injection of the indicated NPs into normal mice. Mice treated with saline were used as the control (*n* = 5). b,c) Expression levels of TNF‐*α*, IL‐6, and IL‐1*β* in the brain (b) and serum (c) were measured by ELISA 24 h after intravenous injection of the indicated NPs into normal mice. d,e) Blood markers of liver function (d) and kidney function (e) were quantitatively measured 24 h after intravenous injection of the indicated NPs into normal mice (*n* = 5). Error bars represent the SD (*n* = 3, unless otherwise specified). ns (not significant), *p < 0.05, **p < 0.01, ***p < 0.001, one‐tailed unpaired *t*‐test.

The pathogenesis of inflammatory diseases is closely associated with the production of proinflammatory cytokines such as TNF‐*α*, IL‐6, and IL‐1*β*.^[^ ^14^, ^32^ ^]^ To evaluate whether the brain accumulation of dOMV@NPs leads to neuroinflammation, mice were treated with either dOMV@NPs or OMV@NPs to measure the intracranial production of proinflammatory cytokines. At both the mRNA and protein levels, both OMV@NPs and LPS significantly increased the intracranial production of TNF‐*α* and IL‐6 (except IL‐1*β* at the protein level), while dOMV@NPs did not cause any significant inflammatory changes (**Figure** **6**a,b). The levels of proinflammatory cytokines in blood were also markedly increased by OMV@NPs and LPS but not dOMV@NPs (Figure 6c). Similar findings were also observed for the levels of proinflammatory cytokines in the liver and spleen (Figure S17, Supporting Information). LPS‐induced acute liver injury and kidney injury are two common complications during sepsis.^[^ ^33^ ^]^ Intravenous injection of OMV@NPs markedly increased plasma levels of alanine transaminase (ALT), aspartate transaminase (AST), blood urea nitrogen (BUN), and creatinine (Cr) at 24 h (Figure 6d). However, dOMV@NPs exhibited extremely low effects on both liver functions and kidney functions, suggesting the prevention of acute hepatotoxicity and nephrotoxicity.

## 3 Conclusion

Biomimetic strategies are often employed in drug delivery, in which drugs are cloaked by endogenous materials (e.g., cell membrane or ligands) to circumvent capture by the reticuloendothelial system or binding to the targeted receptors, thus improving the drug delivery efficiency. There is an urgent and unmet need to design an effective brain‐targeted drug delivery system for both the preclinical and clinical treatment of various brain diseases. To address this problem, many efficient biomimetic drug delivery strategies have been reported. For example, angiopep‐2 peptide‐modified RBCM was developed, with the advantages of improved blood circulation, superb BBB transcytosis, effective tumor accumulation, and specific tumor uptake for glioma delivery of either siRNA or doxorubicin.^[^ ^34^ ^]^ In another study, albumin‐based biomimetic drug delivery systems were developed for BBB penetration and antiglioma therapy based on the upregulated expression of the albumin‐binding proteins SPARC and gp96 on the vessel endothelium of glioma.^[^ ^35^ ^]^ In this study, we addressed the issue of BBB hindrance in brain‐targeted drug delivery from another angle by developing biomimetic EC‐K1/dOMV‐based NPs and successfully demonstrated efficient BBB crossing and brain penetration via OmpA‐gp96 interaction‐mediated transcytosis. The appeal of this system is that the novel EC‐K1‐mimetic drug delivery strategy not only enriches the concept of biomimetic brain‐targeted drug delivery but also increases the brain accumulation rate. Indeed, the involvement of gp96 in the transport of dOMV@NPs through the BBB into the brain parenchyma was verified experimentally and evidenced by efficient in vivo brain accumulation, intracranial microdistribution, and immunohistochemical localization. Most of the dOMV@NPs were found to remain in the intracranial interstitial space. Furthermore, upregulation of gp96 by OmpA‐mediated gp96 signaling may be able to autocatalytically promote the brain‐targeted efficiency of the dOMV@NPs. These results suggest that the dOMV@NPs reported here have the potential to deliver various drugs directly into the brain by crossing the BBB. Based on gp96 expression on brain metastatic cancer cells, the clinical relevance of the present results should be further examined by using animal models of brain metastases. Additionally, the NP diameter directly affects the efficiency of receptor‐mediated transcytosis, suggesting the necessity to further optimize the physicochemical properties of the dOMV@NPs to improve the brain‐targeted efficiency. In addition, the effects of other outer membrane proteins (e.g., NlpI and IbeA^[^ ^36^ ^]^) should be investigated to further elucidate the BBB crossing mechanisms of dOMV@NPs.

## 4 Experimental Section

### Cell Lines and Animals

The bEND.3 mouse BBB endothelial cell line and RAW264.7 macrophage cell line were obtained from the Cell Bank of the Chinese Academy of Sciences, Shanghai, China. The HT22 mouse hippocampal neuronal cell line was kindly provided by Prof. Quanhong Ma at the Institute of Neuroscience, Soochow University. 4T1‐BR5 cells and GFP‐expressed 231Br cells were kindly provided by Dr. Patricia Steeg at the National Cancer Institute. All cells were grown in DMEM supplemented with 10% fetal bovine serum, 100 units mL^−1^ penicillin, and 100 µg mL^−1^ streptomycin in a 37 °C incubator containing 5% CO_2_.

Adult ICR mice (female and 20–25 g body weight), neonatal SD rats (2–3 days), and BALB/c nude mice (female and 18–20 g body weight) were purchased from the Department of Experimental Animals, Soochow University, and maintained under standard housing conditions. All animal experiments were performed in accordance with the guidelines of the Ethics Committee for Animal Experiments and the Soochow University Institutional Animal Care and Use Committee.

### Bacterial Labeling and Intracranial Colonization

E44 (700973, ATCC) is a spontaneous rifampin‐resistant mutant of EC‐K1 RS218 (serotype O18:K1:H7), which is a cerebrospinal fluid isolate from a neonatal infant with meningitis.^[^ ^12^, ^37^ ^]^ The plasmid *pAKgfplux1* was a gift from Attila Karsi (Addgene plasmid # 14083; http://n2t.net/addgene:14083; RRID: Addgene_14083). The plasmid was transferred to E44 by electroporation using a Gene Pulser II system at 2.5 kV, 25 µF, and 400 Ω (Bio–Rad, Hercules, CA).^[^ ^11^ ^]^ Then, bacteria were spread on LB plates containing ampicillin. After incubation at 37 °C for 12 h, ampicillin‐resistant colonies carrying fluorescence and bioluminescence expression plasmids were obtained. The bioluminescence of transformed EC‐K1 cells was investigated using an IVIS Imaging System (Caliper). GFP fluorescence in transformed EC‐K1 cells was detected using flow cytometry (BD FACSCalibur) and fluorescence microscopy (Olympus BX60). To evaluate the intracranial colonization efficiency, neonatal rats and adult mice were intracardially injected with luminous bacteria (1 × 10^3^ CFU and 5 × 10^7^ CFU, respectively) with or without intravenous use of 15 mg kg^−1^ gentamicin. Briefly, neonatal rats and adult mice were anesthetized and firmly secured with front paws extended above the head. EC‐K1 in 0.1 mL PBS 7.4 was loaded into a syringe with a 26‐G needle. After inserting the needle into the second intercostal space 1–2 mm to the left of the sternum at a depth of ≈6 mm, bacteria were injected slowly over 20–30 s. Injected animals were carefully examined and returned to their cages. Then, blood and main organs were collected and imaged to inspect bacterial biodistribution.

### Extraction and Purification of dOMV and OMV

EC‐K1 was cultivated in pH 7.4 LB medium (10 mg mL^−1^ tryptone, 5 mg mL^−1^ yeast extract, and 10 mg mL^−1^ sodium chloride). Both dOMV and OMV were extracted from overnight EC‐K1 cultures (OD_600_ ≈1.5) according to previously described methods.^[^ ^14^ ^]^ For OMV, the supernatant of EC‐K1 culture (obtained by centrifugation at 5000 × *g* at 4 °C for 20 min) was filtered (0.45 µm membrane), concentrated using an Amicon Ultra15 centrifugal filter (100 kDa, Millipore) and further centrifuged at 150 000 × *g* at 4°C for 2 h. Then, the precipitate was resuspended in PBS 7.4 and centrifuged at 150 000 × *g* at 4 °C for another 2 h to finally obtain the OMV pellets.

For dOMV, precipitated EC‐K1 obtained in the process of extracting OMV was resuspended in 1× PBS 7.4 with a volume four times the wet weight of precipitate, homogenized for 30 min by sonication at 300 W in an ice water bath, and transferred to 50‐mL tubes for centrifugation at 2900 × *g* at 4 °C for 60 min. The precipitate was resuspended in 0.1 m Tris buffer (10 × 10^−3^ m EDTA and 5 mg mL^−1^ sodium deoxycholate) with a buffer volume 7.5 times the weight of the wet precipitate to form dOMV and centrifuged at 20 000 × *g* at 4 °C for 1 h to remove the precipitated cell debris. The addition of sodium deoxycholate replaced LPS to form stable dOMV. Then, dOMV in the supernatant were further precipitated at 125 000 × *g* at 4 °C for 2 h twice to finally obtain the dOMV pellets. The dOMV pellets were resuspended in H_2_O or 1× PBS 7.4 for fresh use.

RBCMs were prepared according to a previously described method.^[^ ^38^ ^]^ Briefly, whole blood withdrawn from ICR mice was centrifuged at 800 × *g* at 4 °C for 5 min to carefully remove the serum and the buffy coat. The precipitated red blood cells were rotated in water at 4 °C for 2 h for hemolysis and centrifuged at 12 000 × *g* at 4 °C for 5 min to collect pink RBCM pellets.

### Preparation of dOMV@NPs and OMV@NPs

Parental NPs were prepared using PLGA (acid terminated, molecular weight 38,000‐54,000, Sigma–Aldrich 719900) as the starting material through the emulsion solvent evaporation method as we described previously.^[^ ^13^ ^]^ PLGA with various fluorescence probes in ethyl acetate or dichloromethane was added dropwise to 2.5% PVA‐403 (Kuraray, Japan) under vortexing, sonicated, and then poured into 0.3% PVA‐403, followed by overnight evaporation of ethyl acetate. After centrifugation at 75 940 × *g* at 4 °C for 20 min, the precipitated pellets were collected as parental NPs. To functionalize NPs with various membrane materials, NPs (0.5 mL, 0.5 mg mL^−1^) were mixed with membrane materials (0.5 mL, 0.1 mg mL^−1^) and extruded through a 200 nm polycarbonate filter (Millipore) 11 times to obtain dOMV@NPs, OMV@NPs and RBCM@NPs.

For brain‐targeted A‐NPs, PLGA conjugated with poly(*ε*‐carbobenzoxy‐l‐lysine) (P4510, Sigma) was first synthesized as the starting material to prepare emulsions as we described previously.^[^ ^13^ ^]^ After overnight evaporation of ethyl acetate or dichloromethane, the primary amines from poly(*ε*‐carbobenzoxy‐l‐lysine) at the surface of NPs were reacted with the *N*‐hydroxysuccinimide of maleimide polyethylene glycol succinimidyl carboxymethyl ester (molecular weight 5000, Jenkem, China) for 1 h in PBS 7.4 at room temperature. The exposed maleimide was further reacted with cysteine on angiopep‐2 for 1 h at room temperature. Then, A‐NPs were purified by centrifugation at 75940 × *g* at 4 °C for 20 min and resuspended for fresh use.

### Preparation of Recombinant OmpA and Anti‐OmpA Antibody

Recombinant full‐length OmpA was produced as we described previously.^[^ ^37^ ^]^ Briefly, the gene encoding full‐length OmpA of EC‐K1 was synthesized and cloned into the pMal‐c5x vector to generate pMal‐OmpA. *Escherichia coli* BL21 carrying pMal‐OmpA was induced with 0.5 × 10^−3^ m IPTG for 4 h at 37 °C to express OmpA and sonicated in ice‐cold PBS for 20 min. Then, the supernatant was collected and incubated with amylose resin for 2 h. Finally, OmpA was eluted from the resin with 40 × 10^−3^ m maltose in PBS and loaded on a Resource Q column to remove maltose and endotoxin.

To prepare an anti‐OmpA antibody, BALB/c mice (6–8 weeks old) were immunized with three doses of OmpA at 7‐day intervals. Each injection contained 20 µg OmpA formulated with 25 µg aluminum adjuvant. Seven days after the third immunization, sera were collected.

### Physicochemical Characterization

Appearance and morphology were observed in the presence of phosphomolybdic acid under TEM (HT7700, Japan). SDS–PAGE and western blot were used to characterize all proteins and OmpA, respectively. The secondary antibodies used for western blot are listed in Table S1 (Supporting Information). The hydrodynamic diameter and zeta potential were measured using dynamic light scattering (Zetasizer Nano ZS, Malvern). Endotoxin activity was evaluated using the LAL chromogenic endotoxin quantification kit (60402ES32, Shanghai Yeasen, China) according to the manufacturer's instructions.^[^ ^10^, ^39^ ^]^ For hemolytic toxicity, sheep red blood cells were suspended in normal saline solution to obtain a 10% hematocrit value. Thirty microliters of various samples (2 mg mL^−1^ protein or 224 ng mL^−1^ LPS) were incubated with 0.27 mL red blood cell suspension in a 37 °C water bath for 1 h. The mixture was then centrifuged (625 × *g* at room temperature for 5 min) to collect the supernatant, and the absorbance was measured at 540 nm. The percent hemolysis was determined for each sample by taking the absorbance of water as a 100% hemolytic sample.

### In Vitro BBB Crossing Efficiency and Mechanisms

The effects of various bacterial components (20 µg mL^−1^ protein or 2.24 ng mL^−1^ LPS for 24 h) or NPs (20 µg mL^−1^ protein for 24 h) on the expression of claudin‐5, ZO‐1, and occludin in bEND.3 BBB endothelial cells were characterized by western blot using related antibodies. Original gp96 expression in RAW264.7 macrophages, HT22 neurons, and brain metastatic cancer cells, and the effect of OmpA, dOMV (30 µg mL^−1^ protein for 12 h), and brain metastatic tumor conditioned medium (231Br),^[^ ^13b^ ^]^ on gp96 expression in bEND.3 BBB endothelial cells were characterized by western blot using an anti‐gp96 antibody. The primary antibodies and secondary antibodies used for western blot are listed in Table S1 (Supporting Information).

To measure the effects of various bacterial components on BBB paracellular permeability, compact‐monolayered bEND.3 cells on Transwell inserts with 3.0 µm pores were first treated with bacterial components and then 1 mg mL^−1^ 40 kDa FITC‐dextran (FD40S, Sigma) for 6 h. Afterward, FITC‐dextran in the basolateral chamber was measured at 485/535 nm using a microplate reader (M1000 Pro, Tecan).

For BBB endothelial uptake, bEND.3 cells were treated with the indicated DiR‐labeled NPs (1.5 µg DiR mL^−1^ for 6 h for Figure 3d and 2 µg DiR mL^−1^ for 3 h for Figure S6, Supporting Information). PLGA or PLGA conjugated with poly(*ε*‐carbobenzoxy‐l‐lysine) was mixed with DiR (m/m, 50:1) in ethyl acetate to prepare DiR‐labeled NPs following the procedures described in section Preparation of dOMV@NPs and OMV@NPs. Fresh parental NPs, A‐NPs, and membrane‐coated NPs were mixed with DMSO (*V*_NPs_/*V*_DMSO_, 1:49) to dissolve the NPs. The absorbance of the mixture was measured at 748/780 nm using a microplate reader to quantify the DiR concentration to balance the amount of DiR in the experimental and control groups. For macrophage uptake, RAW264.7 cells were treated with the indicated DiR‐labeled NPs (1.5 µg DiR mL^−1^ for 3 h for Figure 4a and 2 µg DiR mL^−1^ for 3 h for Figure S8, Supporting Information). For uptake by neuronal cells and brain metastatic cancer cells, cells were treated with the indicated DiR‐labeled NPs (2 µg DiR mL^−1^ for 3 h for Figures S13 and S15, Supporting Information). Then, internalized DiR was measured using flow cytometry.

For gp96 localization on BBB endothelial cells, bEND.3 cells were treated with dOMV (60 µg protein mL^−1^ for 1 h) and then immunolabeled with anti‐gp96 antibody and Alexa Fluor 488‐conjugated secondary antibody to visualize gp96 localization under a confocal laser scanning microscope (Nikon AIR HD25). The primary antibodies and fluorescent secondary antibodies used for immunofluorescence staining are listed in Table S1 (Supporting Information).

For competition experiments, bEND.3 cells were first treated with anti‐OmpA antibody (indicated volume of immune serum), anti‐gp96 antibody (indicated amount), or various endocytosis inhibitors (10 µg mL^−1^ chlorpromazine, 25 µg mL^−1^ nystatin, 6.5 mg mL^−1^ M*β*CD, or 100 µg mL^−1^ amiloride) in 1 mL medium and then doxorubicin‐labeled dOMV@NPs (5 µg doxorubicin mL^−1^ for 1.5 h) in the presence of antibodies or various endocytosis inhibitors. Next, internalized doxorubicin was measured using flow cytometry. The primary antibodies used for competition experiments are listed in Table S1 (Supporting Information).

Endosomal escape of dOMV@NPs in BBB endothelial cells was assessed by colocalization of LysoTracker Green DND‐26 and doxorubicin‐labeled dOMV@NPs after internalization. Briefly, cells were incubated with doxorubicin‐labeled dOMV@NPs at 12 µg doxorubicin mL^−1^ for 1 h and then with fresh medium for different times. The treated cells were stained with Hoechst 33342 and LysoTracker Green DND‐26 (40738es50, Shanghai Yeasen, China) and imaged using a confocal laser scanning microscope.

To measure BBB transcellular transport, bEND.3 cells on Transwell inserts were treated with the indicated doxorubicin‐labeled NPs (5 µg doxorubicin mL^−1^ for 6 h). PLGA or PLGA conjugated with poly(*ε*‐carbobenzoxy‐l‐lysine) was mixed with doxorubicin (m/m, 5:1) in ethyl acetate to prepare doxorubicin‐labeled NPs following the procedures described in section Preparation of dOMV@NPs and OMV@NPs.

Fresh parental NPs, A‐NPs, and membrane‐coated NPs were mixed with DMSO (V_NPs_/V_DMSO_, 1:49) to dissolve the NPs. The absorbance of the mixture was measured at 537/584 nm using a microplate reader to quantify the doxorubicin concentration to balance the amount of doxorubicin in the experimental and control groups. Then, doxorubicin in the basolateral chamber was measured at 537/584 nm using a microplate reader. In another experiment, bEND.3 cells on Transwell inserts with or without 231Br cells in the basolateral chamber were treated with doxorubicin‐labeled dOMV@NPs (5 µg doxorubicin mL^−1^ for 6 h) to compare the BBB crossing efficiency under either normal conditions or tumor conditions.

To evaluate the cytotoxicity of doxorubicin‐loaded dOMV@NPs, 231Br cells in 96‐well plates were treated with either free doxorubicin or doxorubicin‐loaded dOMV@NPs at different drug concentrations for 24 h. Then, cell proliferation was quantified using the standard methylthiazolyldiphenyl‐tetrazolium bromide assay. The percentage cell viability of each sample was determined relative to that of the untreated cells.

### In Vivo Pharmacokinetics and Brain Accumulation

For the pharmacokinetic study, free IR780 was dissolved in Cremophor EL and ethanol (v/v, 1:1). Normal mice (*n* = 5) were intravenously injected with either IR780 in Cremophor EL and ethanol or IR780‐loaded dOMV@NPs (0.75 mg IR780 kg^−1^). Blood samples (20 µL) were obtained through the tail vein and mixed with DMSO (180 µL) to extract IR780. Whole mixtures were transferred to 96‐well plates to quantify the near‐infrared fluorescence signal intensity using an IVIS imaging system. The data were analyzed by Living Image 3.0 and compared with standard samples to obtain the blood concentration‐time profiles.

Normal mice (*n* = 3) were intravenously injected with the indicated IR780‐loaded NPs (2.5 µg IR780 per mouse). PLGA or PLGA conjugated with poly(*ε*‐carbobenzoxy‐l‐lysine) was mixed with IR780 (m/m, 50:1) in ethyl acetate to prepare IR780‐loaded NPs following the procedures described in section Preparation of dOMV@NPs and OMV@NPs. Fresh A‐NPs and membrane‐coated NPs were mixed with DMSO (*V*_NPs_/*V*_DMSO_, 1:49) to dissolve the NPs. The absorbance of the mixture was measured at 780/817 nm using a microplate reader to quantify the IR780 concentration to balance the amount of IR780 in the experimental and control groups. At 8 and 24 h after injection, the mice were perfused with PBS 7.4 and 2% paraformaldehyde. Then, fixed brains were excised and imaged using an IVIS imaging system. The IR780 fluorescence intensity in the brain was semiquantified using Living Image 3.0.

To further quantify the brain accumulation of dOMV@NPs using ICP–MS, PLGA, or PLGA conjugated with poly(*ε*‐carbobenzoxy‐l‐lysine) was mixed with SPIO (m/m, 5:1) in dichloromethane to prepare SPIO‐loaded NPs,^[^ ^24^ ^]^ following the procedures described in section Preparation of dOMV@NPs and OMV@NPs.

Before injection, the iron concentrations of A‐NPs and membrane‐coated NPs were analyzed using ICP–MS (Thermo Scientific Element 2) to balance the amount of SPIO in the experimental and control groups. Then, normal mice (*n* = 5) were intravenously injected with the indicated SPIO‐loaded NPs (5 mg Fe kg^−1^ mouse weight). For a control group, mice were intravenously injected with an anti‐gp96 antibody (15 µg in 100 µL) before the injection of SPIO‐loaded dOMV@NPs. After 8 h, the mice were perfused with PBS 7.4, and the brains were excised, mixed with nitric acid, digested at 200 °C in a microwave oven for 30 min, diluted with ultrapure water, and analyzed with ICP–MS to quantify the iron concentration.

To quantify brain accumulation using the percentage of injected dose, normal mice (*n* = 5) were intravenously injected with IR780‐loaded dOMV@NPs (2.5 µg IR780 per mouse) and perfused with PBS 7.4 at 8 h and 24 h after injection. Then, the brains were excised, homogenized, and infiltrated with DMSO (9 times the wet brain weight) to extract IR780. Extracted IR780 was measured at 780/817 nm using a microplate reader.

### Intracranial Microdistribution and Immunohistochemical Localization

Freshly prepared doxorubicin‐labeled dOMV@NPs were stored at room temperature for different times to evaluate the stability of doxorubicin fluorescence. Then, normal mice were intravenously injected with the indicated doxorubicin‐labeled NPs at a dose of 5 mg doxorubicin kg^−1^ twice at intervals of 12 h. At 12 h after injection, mice were perfused with PBS 7.4 and 2% paraformaldehyde. Excised brains were further fixed in 2% paraformaldehyde at 4 °C for 6 h and then sequentially soaked in 15 wt.% and 30 wt.% sucrose at 4 °C. The dehydrated specimens were frozen in optimal cutting temperature compound and then sliced into 20‐µm‐thick sections using a CM1950 cryostat (Leica Microsystems, Wetzler, Germany). Next, the sections were stained with DAPI in an antifade mounting medium to visualize the nuclei and imaged for doxorubicin using a confocal laser scanning microscope to study intracranial microdistribution.

To evaluate intracranial immunohistochemical localization, cerebral sections for various intracranial regions were immunolabeled with antibodies against CD31 (1:100), GFAP (1:300), Iba1 (1:500), and NeuN (1:1000) to visualize BBB endothelial cells, astrocytes, microglia, and neurons, respectively. Next, the sections were incubated with an Alexa Fluor 488‐conjugated secondary antibody and imaged using a confocal laser scanning microscope. The primary antibodies and fluorescent secondary antibodies used for immunofluorescence staining are listed in Table S1 (Supporting Information).

### In Vivo Targeting Efficiency and Therapeutic Efficacy of Doxorubicin‐Loaded dOMV@NPs in Mice with Brain Metastases

To establish an animal model of brain metastases, nude mice were anesthetized and firmly secured with front paws extended above the head. Approximately 2.5 × 10^5^ 231Br cells in 0.1 ml PBS 7.4 were loaded into a syringe with a 26‐G needle. After inserting the needle into the third intercostal space, 3 mm to the left of the sternum at a depth of ≈6 mm, cells were injected slowly over 20–30 s. Then, the mice were carefully checked and returned to their cages.

Mice with 231Br brain metastases were intravenously injected with doxorubicin‐loaded dOMV@NPs at a dose of 5 mg doxorubicin kg^−1^ twice at intervals of 12 h after the onset of neurological symptoms. Twelve hours after the second injection, the mice were perfused and fixed. Then, the brain was harvested, dehydrated, and sectioned into 20 µm thick sections. Sections without GFP‐expressed 231Br cells (normal brain regions) were stained to label astrocytes, microglia, and neurons, respectively. The sections were examined under a confocal laser scanning microscope.

In vivo treatments for evaluating the therapeutic efficacy were started 5 days after intracardiac injection of 231Br cells. Mice were given saline, free doxorubicin, or doxorubicin‐loaded dOMV@NPs (*n* = 8). Treatments were administered through the tail vein twice a week at a dose of 5 mg doxorubicin kg^−1^. Mice were monitored for survival until one of the following criteria for euthanasia was met: 1) the body weight of the mouse dropped by 15% of its initial weight, and 2) the mouse became lethargic or sick and unable to feed.

### Effects on Physiological Function and the Immune System

To evaluate whether the brain accumulation of dOMV@NPs would lead to neuroinflammation, normal mice (*n* = 5) were intravenously injected with either dOMV@NPs or OMV@NPs (6.86 mg protein kg^−1^). Intracranial production of proinflammatory cytokines was measured at 24 h after injection. Briefly, total RNA was isolated from perfused brains using TRIzol reagent (15596018, Invitrogen) according to the manufacturer's protocols. Then, 1 µg total RNA was used for cDNA first‐strand synthesis using a reverse transcriptase kit (K1622, Thermo Fisher, USA). Quantitative PCR was performed to determine the transcriptional levels of TNF‐*α*, IL‐6, and IL‐1*β* using 2× SYBR Green PCR Master Mix (B21702, Bimake, China) in an Applied Biosystems 7500 real‐time PCR system. GAPDH was used as an internal reference. Relative gene expression levels were determined via the 2^−ΔΔCt^ method. All the mouse‐specific primers for real‐time PCR are listed in Table S2 (Supporting Information).

To evaluate the production of proinflammatory cytokines at the protein level, normal mice (*n* = 3) were intravenously injected with dOMV@NPs, OMV@NPs (6.86 mg protein kg^−1^), or LPS (0.823 µg kg^−1^). At 24 h after injection, the expression of TNF‐*α*, IL‐6, and IL‐1*β* in the serum and perfused brain, liver, and spleen was measured using ELISA. Homogenized tissues in PBS 7.4 with 1% PMSF were centrifuged at 12 000 × *g* at 4°C for 15 min to remove all insoluble materials. The levels of TNF‐*α*, IL‐6, and IL‐1*β* in the serum and supernatants of tissue homogenates were measured by ELISA kits according to the manufacturer's instructions (EK282/4‐96, EK206/3‐96, EK201BHS‐96, Hangzhou MultiSciences, China).

To evaluate LPS‐induced acute liver injury and kidney injury, normal mice (*n* = 5) were intravenously injected with either dOMV@NPs or OMV@NPs (6.86 mg protein kg^−1^). Blood samples were collected using the submandibular bleeding method at 24 h after injection and centrifuged at 625 × *g* at 4 °C for 10 min to separate the plasma, which was then subjected to ALT, AST, BUN, and Cr testing using commercial detection kits (C009‐2, C010‐2, C013‐2‐1, C011‐2‐1, Nanjing Jiancheng, China) according to the manufacturer's instructions. Saline‐treated mice were used as controls.

### Statistical Analysis

All quantitative data are presented as the mean ± standard deviation (SD). One‐tailed unpaired Student's *t*‐test or log‐rank (Mantel‐Cox) test was performed to determine statistical significance. Log‐rank (Mantel‐Cox) test was used to compare treatment groups in the survival study. Unpaired Student's *t*‐test was used for other studies. All statistical tests were performed in GraphPad Software (Prism 8.0.1). ns means not significant, *p* < 0.05 (*), 0.01 (**), and 0.001 (***) were considered significant.

## Conflict of Interest

The authors declare no conflict of interest.

## Supporting information

Supporting Information

## Data Availability

Research data are not shared.
