# Supplementary material for: Biomimetic Lipopolysaccharide‐Free Bacterial Outer Membrane‐Functionalized Nanoparticles for Brain‐Targeted Drug Delivery
Source: Adv Sci (Weinh). 2022 Mar 31;9(16):2105854. doi: 10.1002/advs.202105854 (PMC9165477; doi:10.1002/advs.202105854)
Supplement: Supplementary file 1 — Supporting Information [file ADVS-9-2105854-s001.pdf]

ADVANCED SCIENCE
Open Access

Supporting Information

for *Adv. Sci.*, DOI 10.1002/advs.202105854

Biomimetic Lipopolysaccharide-Free Bacterial Outer Membrane-Functionalized Nanoparticles for Brain-Targeted Drug Delivery

*Haiyan Chen, Mengyuan Zhou, Yuteng Zeng, Tongtong Miao, Haoyuan Luo, Yang Tong, Mei Zhao, Rui Mu, Jiang Gu, Shudi Yang and Liang Han**

Supporting Information

Biomimetic lipopolysaccharide-free bacterial outer membrane-functionalized nanoparticles for brain-targeted drug delivery

*Haiyan Chen, Mengyuan Zhou, Yuteng Zeng, Tongtong Miao, Haoyuan Luo, Yang Tong, Mei Zhao, Rui Mu, Jiang Gu, Shudi Yang, and Liang Han**

Supporting Information includes:

Figure S1 to S17.

Table S1 and S2.

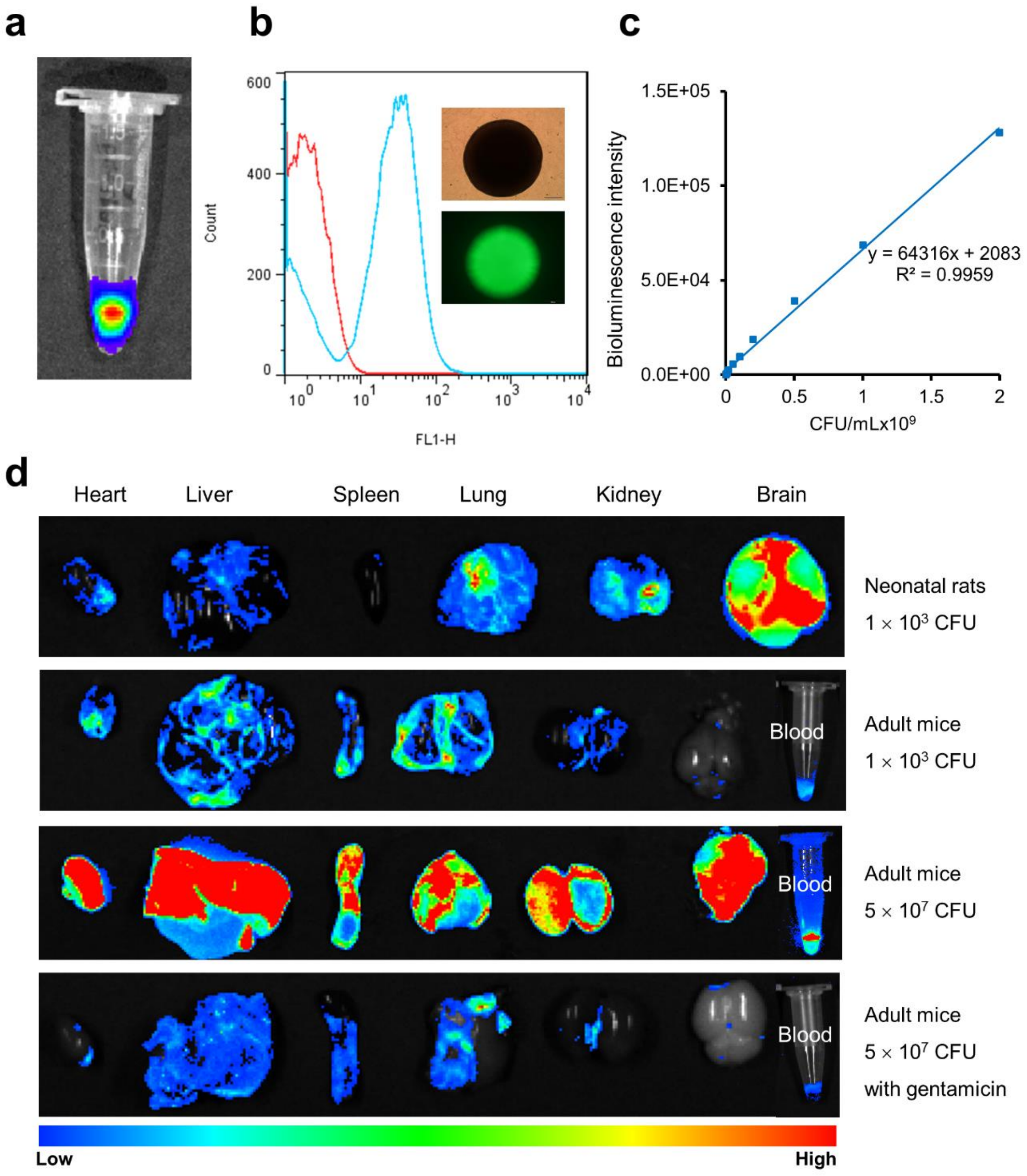

Figure S1. Active EC-K1 was observed in the brains of both neonatal rats and adult mice. (a) Bioluminescence images of active EC-K1 transformed with plasmid *pAKgfplux1* were obtained using an IVIS Imaging System. (b) Flow cytometry analysis of wild-type EC-K1 (red) and transformed EC-K1 (light green). Insets show the bright field image and fluorescence image of transformed EC-K1. (c) A linear relationship exists between the bioluminescence intensity and colony-forming units (CFU) of transformed EC-K1 cells. (d) *In vivo* biodistribution of transformed EC-K1 in neonatal rats and adult mice with or without intravenous administration of 15 mg kg^{-1} gentamicin. Neonatal rats and adult mice were given intracardiac injections of 1×10^3 CFU- and 5×10^7 CFU-transformed EC-K1 cells, respectively. Organs and blood were collected at 12 h after injection for bioluminescence imaging.

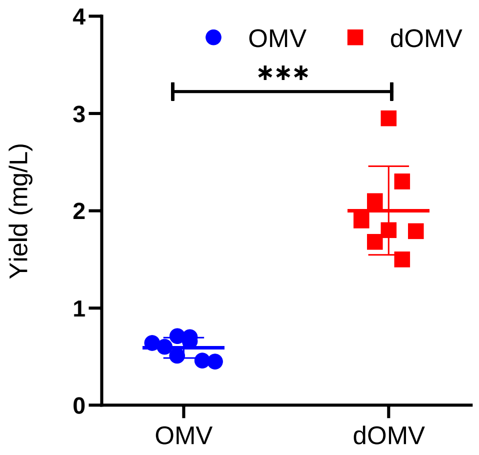

Figure S2. The yield of OMV and dOMV from EC-K1 overnight cultures (OD_{600} ~1.5), expressed as protein amount per liter culture. Error bars represent the SD ($n = 8$). ***$p < 0.001$, one-tailed unpaired t test.

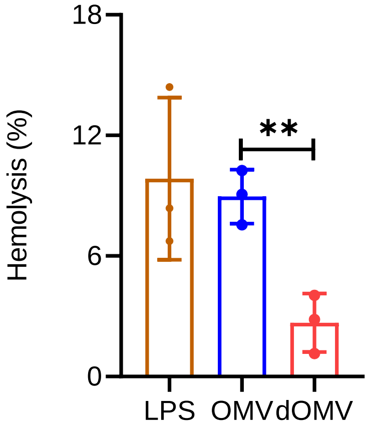

Figure S3. Hemolytic behaviors of the indicated treatments (2 mg ml^{-1} protein or 224 ng ml^{-1} lipopolysaccharide). Water was used for 100% hemolysis, and saline was used for zero hemolysis. Error bars represent the SD ($n = 3$). **$p < 0.01$, one-tailed unpaired t test.

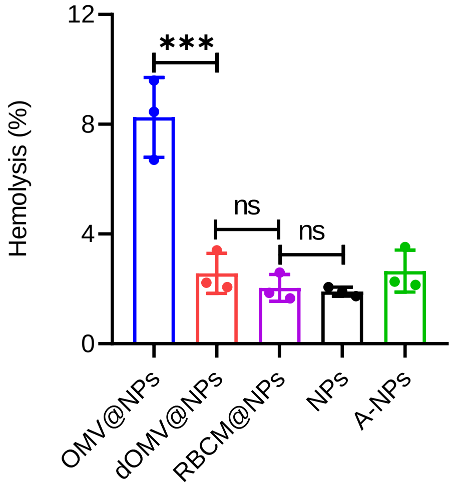

Figure S4. Hemolytic behaviors of the indicated NPs (2 mg ml^{-1} protein). Water was used for 100% hemolysis, and saline was used for zero hemolysis. Error bars represent the SD (n = 3). ns (not significant), ***$p < 0.001$, one-tailed unpaired t test.

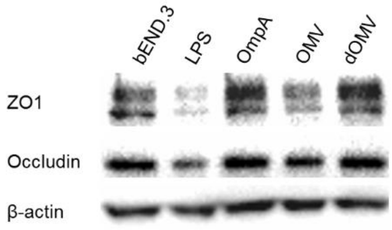

Figure S5. Levels of ZO-1 and occludin on bEND.3 BBB endothelial cells after stimulation with the indicated bacterial components (20 μg ml^{-1} protein or 2.24 ng ml^{-1} LPS for 24 h) were analyzed by western blot.

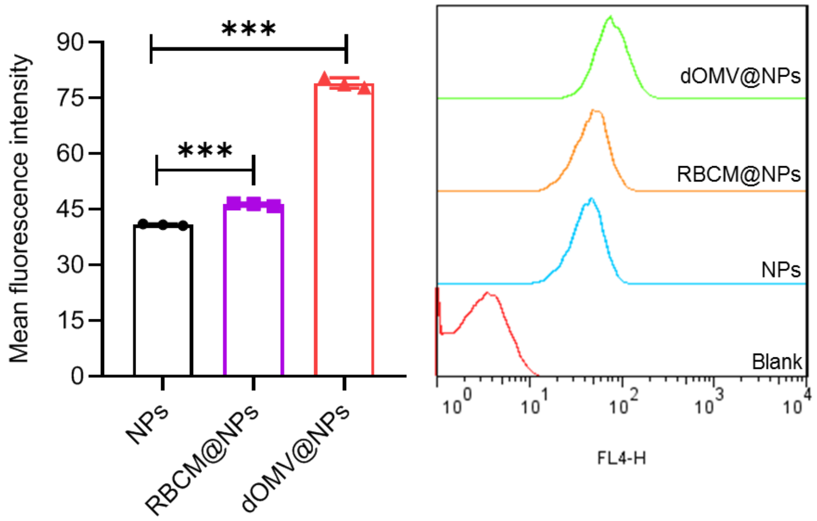

Figure S6. *In vitro* uptake of the indicated DiR-labeled NPs (2 μg DiR ml^{-1} for 3 h) by bEND.3 cells. Error bars represent the SD ($n = 3$). ***$p < 0.001$, one-tailed unpaired t test.

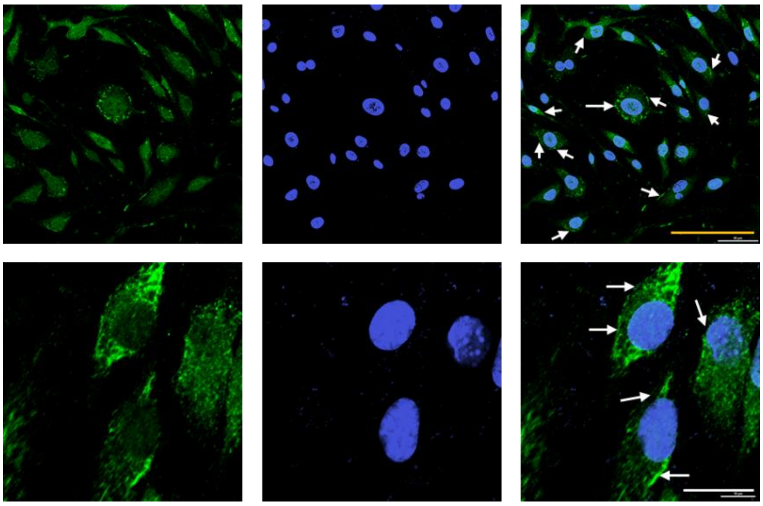

Figure S7. gp96 localization (green) in bEND.3 cells pretreated with dOMV (60 μg protein ml^{-1} for 1 h). Nuclei (blue) were stained with DAPI. White arrows indicate localization at the cell membrane. The yellow scale bar and white scale bar indicate 100 μm and 20 μm, respectively.

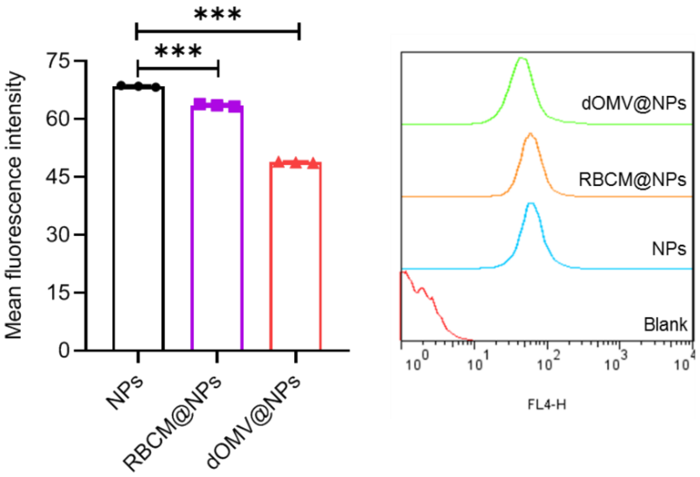

Figure S8. *In vitro* uptake of the indicated DiR-labeled NPs (2 μg DiR ml^{-1} for 3 h) by RAW264.7 cells. Error bars represent the SD ($n = 3$). ***$p < 0.001$, one-tailed unpaired t test.

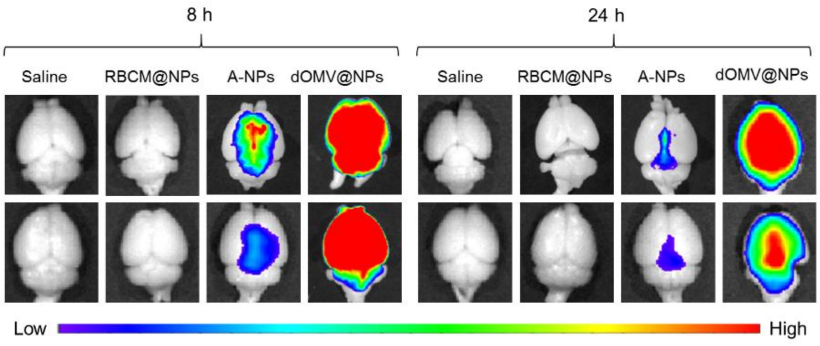

Figure S9. Qualitative images of brain accumulation of IR780 in normal mice at 8 h and 24 h after injection of the indicated IR780-labeled NPs.

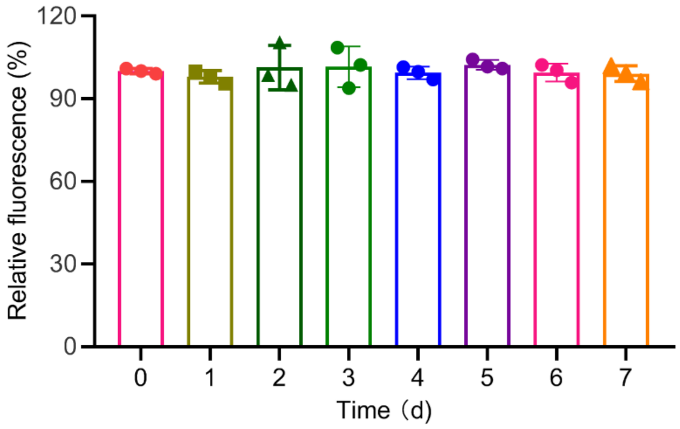

Figure S10. The fluorescence stability of doxorubicin-labeled dOMV@NPs is shown as a percentage relative to the fluorescence intensity of freshly prepared doxorubicin-labeled dOMV@NPs. Error bars represent the SD (n = 3).

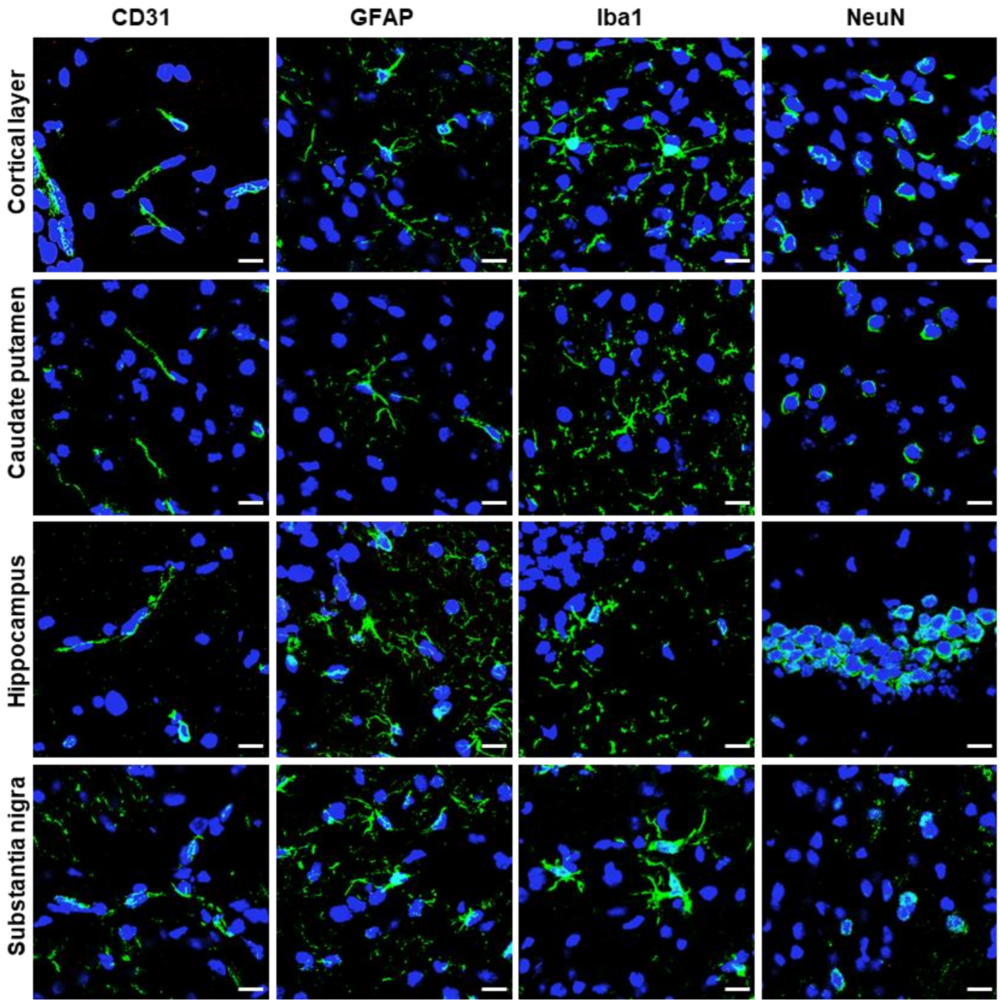

Figure S11. Immunohistochemical analysis of cerebral sections for the indicated intracranial regions was performed to check the colocalization of RBCM@NPs with various types of intracranial cells. Cerebral sections obtained at 12 h after the second intravenous injection of doxorubicin-labeled RBCM@NPs were stained with anti-CD31, anti-GFAP, anti-Iba1, and anti-NeuN antibodies (green). Nuclei (blue) were stained with DAPI. Scale bars indicate 20 μm.

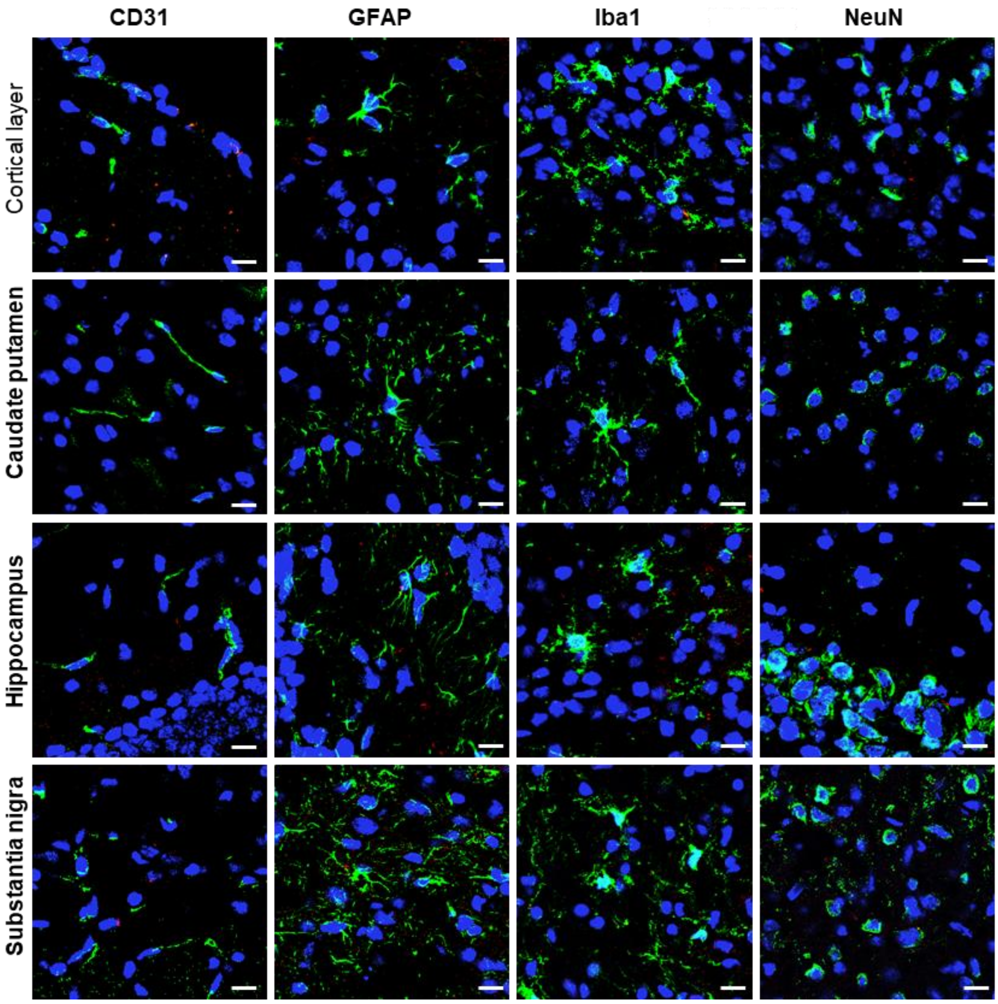

Figure S12. Immunohistochemical analysis of cerebral sections for the indicated intracranial regions was performed to check the colocalization of A-NPs with various types of intracranial cells. Cerebral sections obtained at 12 h after the second intravenous injection of doxorubicin-labeled A-NPs were stained with anti-CD31, anti-GFAP, anti-Iba1, and anti-NeuN antibodies (green). Nuclei (blue) were stained with DAPI. Scale bars indicate 20 μm.

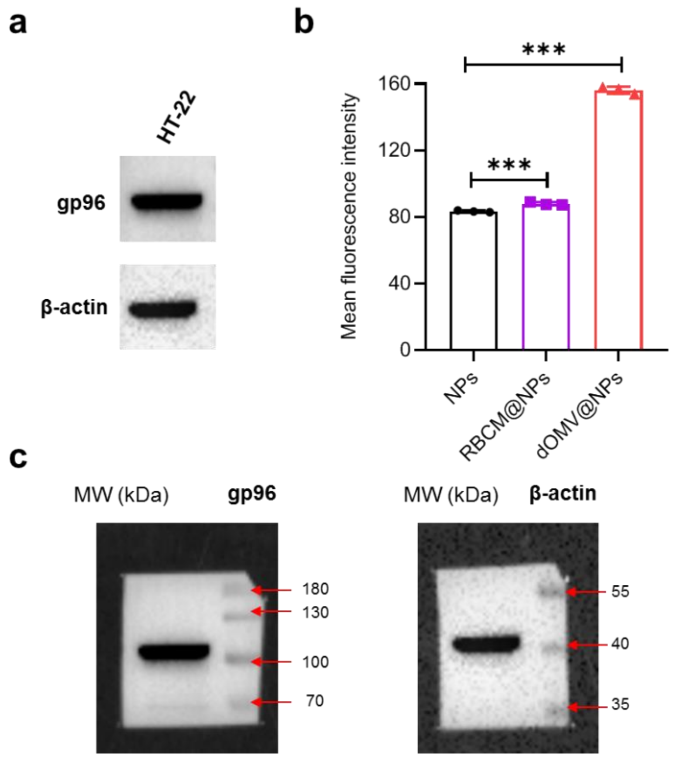

Figure S13. gp96 expression and cellular uptake in HT22 neurons. (a) gp96 expression in HT22 cells was characterized by western blot. (b) *In vitro* uptake of the indicated DiR-labeled NPs (2 μg DiR ml^{-1} for 3 h) by HT22 neurons. (c) Full scans of western blot analysis in (a). MW: molecular weight. Error bars represent the SD ($n = 3$). ***$p < 0.001$, one-tailed unpaired t test.

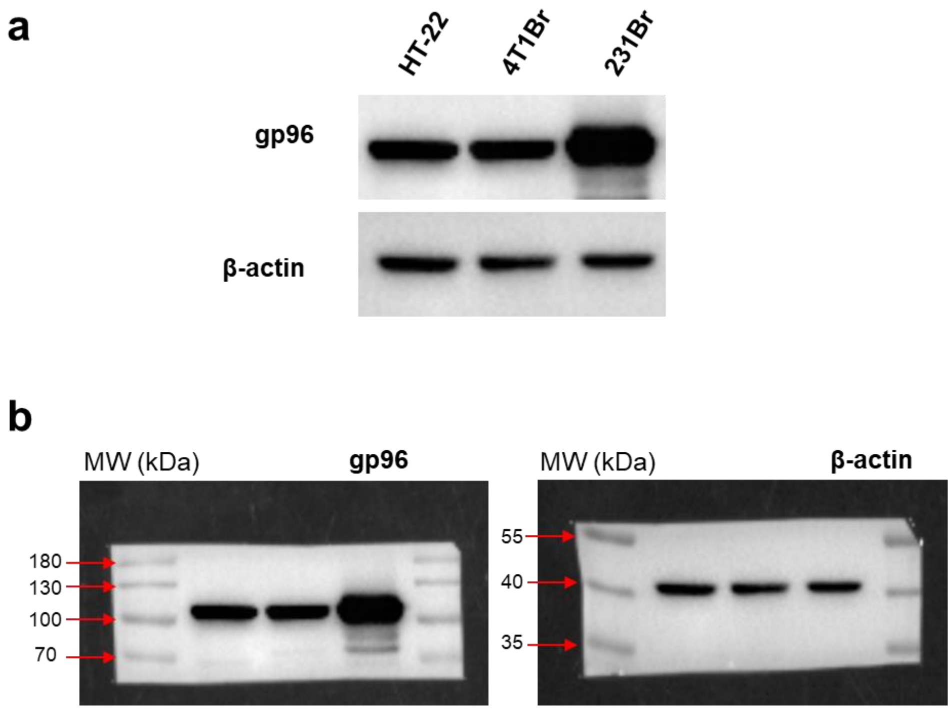

Figure S14. (a) gp96 expression in HT22, 4T1Br and 231Br cells was characterized by western blot. (b) Full scans of western blot analysis in (a). MW: molecular weight.

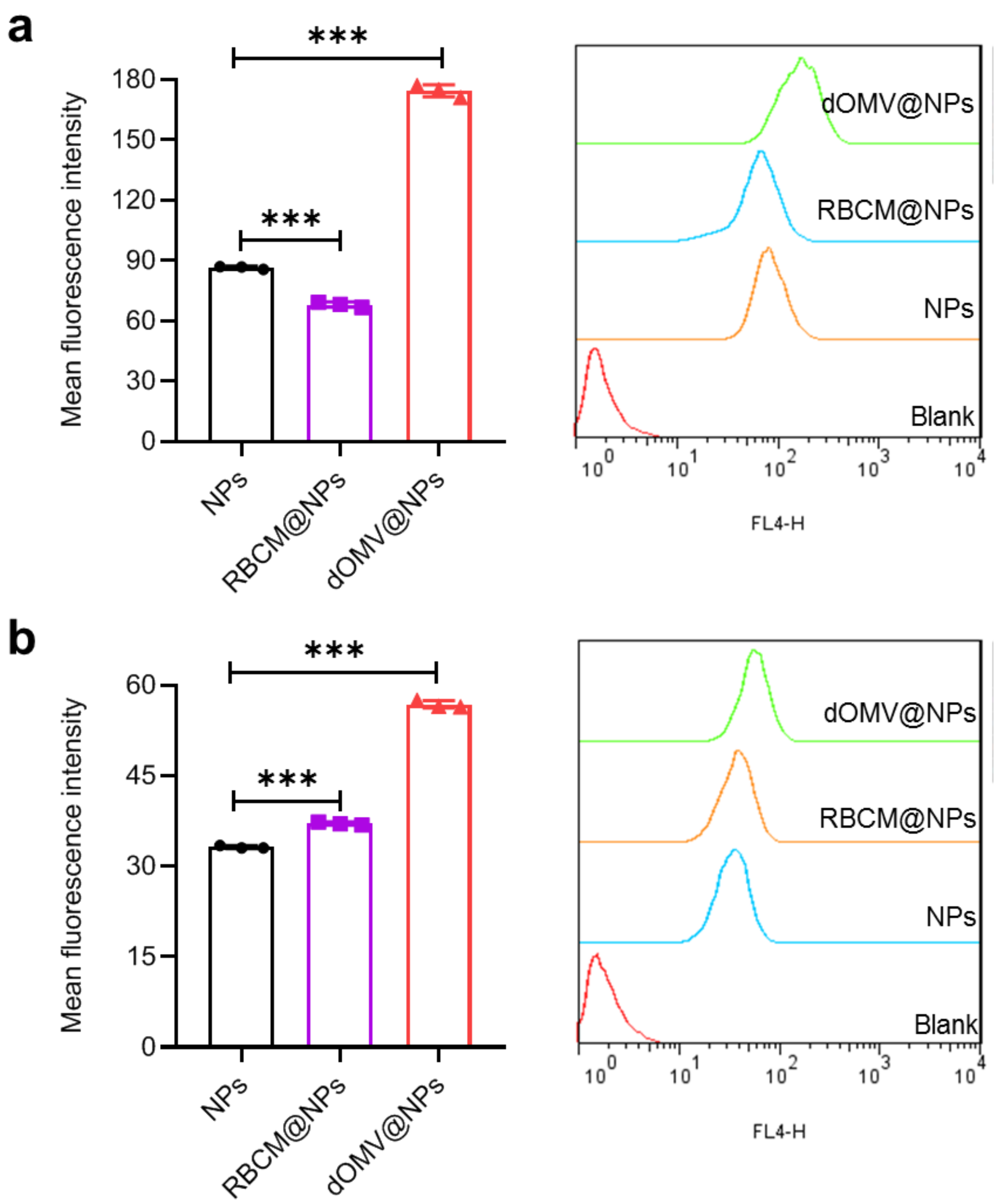

Figure S15. (a, b) *In vitro* uptake of the indicated DiR-labeled NPs (2 μg DiR ml^{-1} for 3 h) by 231Br cells (a) and 4T1-BR5 cells (b). Error bars represent the SD (n = 3). ***$p < 0.001$, one-tailed unpaired t test.

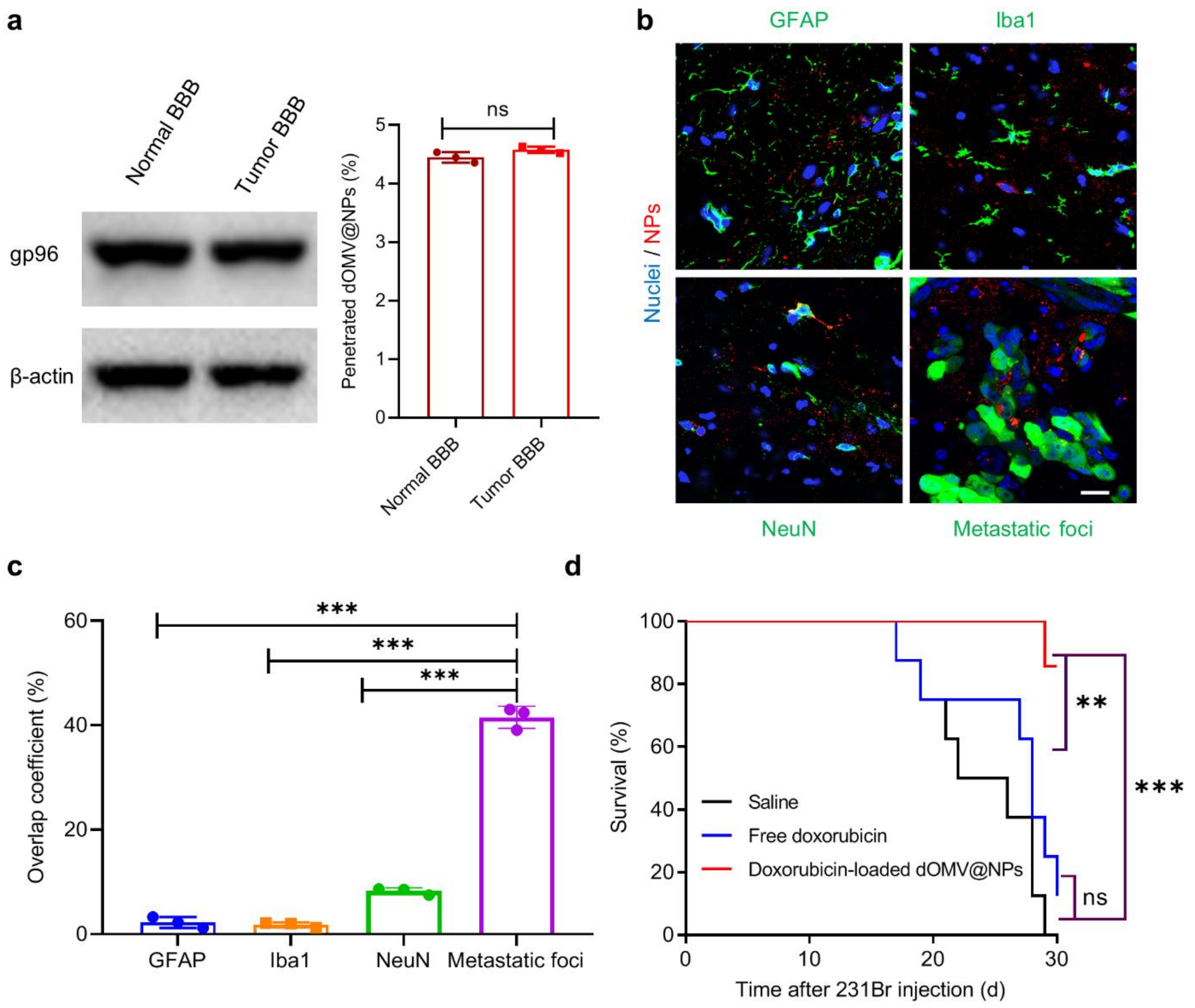

Figure S16. (a) The expression of gp96 in bEND.3 cells cultured in normal medium (normal BBB) or 231Br conditioned medium (tumor BBB) was analyzed by western blot. Penetration of doxorubicin-labeled NPs (5 μg doxorubicin ml^{-1} for 6 h) across the *in vitro* normal BBB or tumor BBB. Error bars represent the SD (n = 3). ns (not significant), one-tailed unpaired t test. (b) Immunohistochemical analysis of cerebral sections (obtained at 12 h after the second injection) from mice bearing 231Br brain metastases after treatment with doxorubicin-loaded dOMV@NPs. The 231Br cells stably express GFP. Sections without GFP (normal brain regions) were stained to label astrocytes, microglia, and neurons. Red, doxorubicin-loaded dOMV@NPs; green, various types of intracranial cells; blue, DAPI. Scale bar indicates 20 μm. (c) The overlap coefficient between doxorubicin and brain cells. Error bars represent the SD (n = 3). ***$p < 0.001$, one-tailed unpaired t test. (d) Kaplan–Meier survival curves for mice bearing brain metastases with the indicated treatments (n = 8). ns (not significant), **$p < 0.01$, ***$p < 0.001$, log-rank (Mantel-Cox) test.

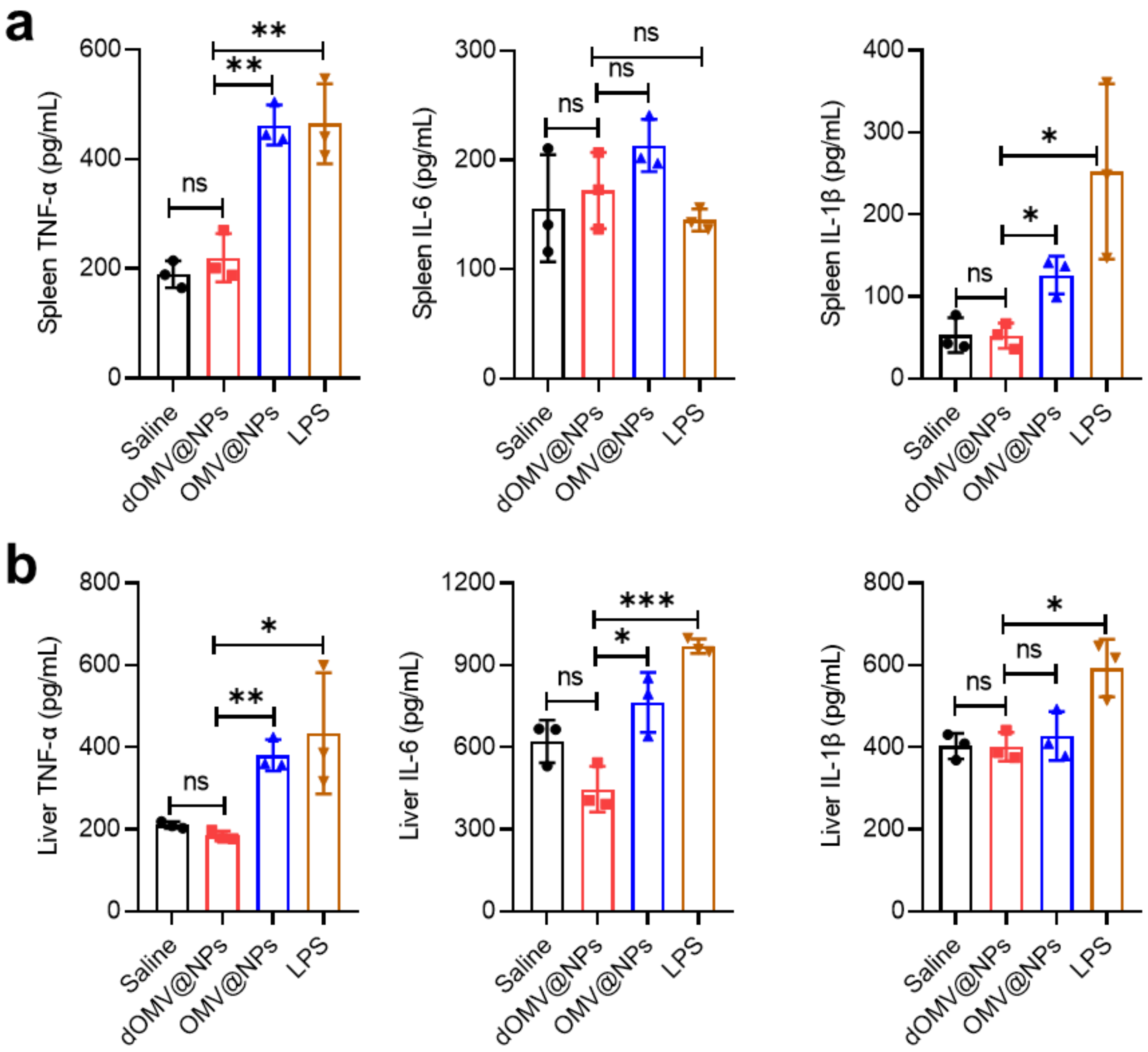

Figure S17. (a, b) Expression levels of TNF-α, IL-6, and IL-1β in spleens (a) and livers (b) were measured by ELISA at 24 h after intravenous injection of the indicated NPs into normal mice. Error bars represent the SD (n = 3). ns (not significant), *$p < 0.05$, **$p < 0.01$, ***$p < 0.001$, one-tailed unpaired t test.

Supplementary Tables

Supplementary Table 1. Antibodies used in the study.

Antibodies	Company	Catalog No.	Application	Dilution fold
Anti-gp96	Proteintech	14700-1-AP	Competitive	200, 1000
Anti-OmpA	Prepared in the study	/	Competitive	33, 83, 133
Anti-OmpA	Prepared in the study	/	WB	1000
Anti-gp96	Proteintech	10979-1-AP	WB, IF	1500, 200
Anti-Claudin-5	Invitrogen	341600	WB	200
Anti-CD31	Abcam	ab28364	IF	100
Anti-GFAP	CST	3670s	IF	300
Anti-Iba1	Abcam	ab178846	IF	500
Anti-NeuN	Abcam	ab104224	IF	1000
Anti-ZO1	Abcam	ab276131	WB	1000
Anti-Occludin	Abcam	ab216327	WB	1000
Anti-β-actin	Beyotime	AF5001	WB	8000
HRP-labeled goat anti-mouse IgG(H+L)	Beyotime	A0216	WB for OmpA	1000
HRP-labeled goat anti-rabbit IgG(H+L)	Beyotime	A0208	WB for gp96 and Claudin-5	1000
Alexa Fluor 488 goat anti-mouse IgG(H+L)	Beyotime	A0428	IF for NeuN and GFAP	500
Alexa Fluor 488 goat anti-rabbit IgG(H+L)	Beyotime	A0423	IF for gp96, CD31 and Iba1	500

WB: western blot. IF: immunofluorescence.

Supplementary Table 2. Primers used in this study.

Primer	Primer sequence, 5'-3'
TNF-α (forward)	CTGAGGTCAATCTGCCCAAGTAC
TNF-α (reverse)	CTTCACAGAGCAATGACTCCAAAG
IL-6 (forward)	GAGGATACCACTCCCAACAGACC
IL-6 (reverse)	AAGTGCATCATCGTTGTTCATACA
IL-1β (forward)	CTAAAGTATGGGCTGGACTG
IL-1β (reverse)	AGCTTCAATGAAAGACCTCA
GAPDH (forward)	GAAGGTCGGTGTGAACGGAT
GA PDH (reverse)	AATCTCCACTTTGCCACTGC
